# Elevated Siglec-7 expression correlates with adverse clinicopathological, immunological, and therapeutic response signatures in breast cancer patients

**DOI:** 10.3389/fimmu.2025.1573365

**Published:** 2025-06-06

**Authors:** Hamza Benthami, Basma Zohair, Ibtissam Rezouki, Oumayma Naji, Kenza Miyara, Simohamed Ennachit, Mohamed Elkarroumi, Hind Aschawa, Abdallah Badou

**Affiliations:** ^1^ Immuno-Genetics and Human Pathologies Laboratory (LIGEP), Faculty of Medicine and Pharmacy, Hassan II University, Casablanca, Morocco; ^2^ Department of Obstetrics and Gynecology, Mohamed VI Oncology Center, University Hospital Center (CHU) Ibn Rochd, Casablanca, Morocco; ^3^ Department of Nuclear Medicine, University Hospital Center (CHU) Ibn Rochd, Casablanca, Morocco

**Keywords:** Siglec-7, A2AR, tumor microenvironment, immunosuppression, immune checkpoint, immunotherapy, breast cancer prognosis, cancer therapy

## Abstract

**Introduction:**

Breast cancer is a highly heterogeneous malignancy, characterized by an intricate, hypersialylated tumor microenvironment that actively inhibits immune functions. Although immune checkpoint inhibitors have marked clinical advantages in various tumor types, their efficacy in Triple-Negative Breast Cancer (TNBC) and Human Epidermal Growth Factor Receptor 2 positive (HER2+) patients remains limited. Siglec-7 has emerged as a novel and promising candidate for advancing cancer immunotherapy.

**Methods:**

Herein, we explored the potential role of Siglec-7, a key inhibitory glycoimmune checkpoint, in human breast tumor microenvironment in a cohort of 45 Moroccan patients. Subsequently, data were corroborated using large-scale data from independent cohorts of American and British-Canadian patients, the TCGA and METABRIC.

**Results:**

We report that Siglec-7 transcripts were significantly upregulated in breast tumor tissues compared to matched adjacent non-invaded tissues. Furthermore, higher Siglec-7 expression correlated with poor clinicopathological features, including negative Estrogen Receptor (ER) and Progesterone Receptor (PR) status, advanced tumor grades, and unfavorable patient prognosis. Siglec-7 overexpression was linked to gene expression patterns, known to promote breast tumor progression through pathways involved in proliferation, invasion, and immune escape. Notably, high Siglec-7 expression was associated with increased infiltration of immunosuppressive cells and T-cells with an exhausted phenotype. Interestingly, a positive correlation between siglec-7 and A2AR, another emerging and potent inhibitory checkpoint, was revealed, along with the co-upregulation of other inhibitory immune checkpoint genes; and this was associated with signatures of impaired immune effector functions. Finally, our data also pinpointed an interesting role of high Siglec-7 expression in predicting resistance to conventional therapies, including chemotherapy, endocrine treatments, and immunotherapy.

**Discussion:**

These findings strongly suggest a central role for Siglec-7 as a promising therapeutic target and prognostic biomarker for human breast cancer, warranting further investigation into its clinical potential.

## 1 Introduction

Breast cancer remains the second most frequently diagnosed cancer, with 2,296,840 new cases, and ranks fourth in cancer-related mortality, accounting for 666,103 deaths annually, reflecting its significant health burden worldwide (1). This pathology is distinguished by notable inter- and intratumoral heterogeneity, encompassing numerous clinicopathological subtypes that exhibit genetic and phenotypic diversity, contributing to varied therapeutic outcomes (2). Triple Negative Breast Cancer (TNBC), which accounts for 15-20% of cases, is particularly aggressive, defined by the absence of estrogen (ER), progesterone (PR) receptors, and the epidermal growth factor receptor 2 (HER2) (3). Chemotherapy, a key component of standard breast cancer treatments, is commonly recommended for this molecular subtype, while hormonotherapy and targeted therapies are specifically tailored for receptor-positive breast cancers, including the Luminal and HER2+ subtypes (4). Clinically, TNBC and HER2+ cancers present a significantly higher risk of early metastasis, recurrence, and poor survival, with resistance to conventional treatment. This resistance is likely due to processes that extend beyond genetic and epigenetic alterations, particularly involving the tumor microenvironment (TME) (5–10).

Current understanding of this cancer ecosystem recognizes the immune component as a pivotal factor in determining patient outcomes. This has led to substantial advancements in treatment strategies, especially with the approval of immune checkpoint inhibitors (ICIs) for various cancers (11). Through the activation of inhibitory immune checkpoints, classically PD-1, PD-L1, and CTLA-4, tumors disrupt the immune system homeostatic balance, thereby inducing immune suppression that enables the evasion of immune surveillance and promotes tumor progression (12). Although targeting this immune escape mechanism with ICIs has proven beneficial in other cancers, these treatments have regrettably shown limited effectiveness in patients with TNBC and HER2+ subtypes, as evidenced by clinical trials (7, 13–15). Consequently, the necessity for further research becomes evident to better comprehend breast TME, a critical factor in refining immunotherapy approaches for these patients (11).

Beyond classical immune suppression mechanisms, hypersialylation emerges as a novel immune checkpoint pathway and a key orchestrator of immune evasion, playing a pivotal role in tumorigenesis (16, 17). Indeed, a growing body of evidence associates sialic acid accumulation not only with the promotion of immunosuppression but also with the disruption of critical regulatory signaling pathways, ultimately diminishing the efficacy of chemotherapy and radiotherapy (18–20). Interestingly, aggressive subtypes of breast cancer exhibit markedly higher levels of aberrant hypersialylation compared to both normal tissues and favorable prognosis molecular subtypes, underscoring its significance in the disease pathophysiology (21).

Mechanistically, sialic acids engage with immune cells via recognition by glycoimmune checkpoints, a family of molecules known as sialic acid-binding immunoglobulin-like lectins (SIGLECs) (22). SIGLECs, expressed on various immune cells, have garnered increasing interest as potential targets for modulating the immune response and enhancing antitumor activity (23). This family in humans consists of 14 members, classified into two categories based on sequence similarity, namely the conserved SIGLECs and the rapidly evolving CD33-related SIGLECs (24). Among the members of the latter group, Siglec-7, also referred to as p75/AIRM1 and CD328, represents the seventh member of the SIGLEC family and has recently attracted considerable focus in cancer immunology (25). Siglec-7 is specifically expressed on key immune cells involved in the antitumoral immune response, including natural killer (NK) cells, myeloid cells, and T lymphocytes, thereby distinguishing it from other Siglecs (26–28). Featuring an extracellular domain for sialic acid binding, this receptor is characterized by an intracellular immunoreceptor tyrosine-based inhibitory motif (ITIM), which mediates immune cell inhibition upon ligand engagement (24). Indeed, Siglec-7 plays a significant role in intra-immune signaling regulation as evidenced by increased activation of both primary T-cells and antigen-presenting dendritic cells *in vitro* following its blockade (29, 30). In pancreatic ductal adenocarcinoma, elevated sialylation correlates with increased infiltration of tumor-associated macrophages, originating from monocytes expressing Siglec-7 and Siglec-9, contributing to a poor prognosis (31). Additionally, increased immune cell infiltration and reduced tumor growth were observed following the blockade of the Siglec-7/9–sialic acid interaction in a humanized mouse model of prostate cancer (32). Strikingly, Siglec-7 expression was upregulated on tumor-infiltrating CD8+ T cells in both colon cancer tissues and ovarian cancer, and was linked to the expression of coinhibitory checkpoints such as PD-1 and LAG-3 (30). Furthermore, treating NK cells with Siglec-7 antibodies in a co-culture model of ovarian cancer cell lines led to a marked increase in their cytotoxic activity, strengthening NK-mediated antitumor response (33). In the context of breast cancer, recent research has linked the expression of Siglec-7 on myeloid cells to fibrosis pathogenesis (21). However, a comprehensive understanding of Siglec-7 expression and role in human breast cancer, as well as in-depth correlations with clinicopathological parameters and links to immunological, prognostic, and therapeutic response signatures, remains elusive and warrants further investigation.

In this study, we aimed to address this research gap by assessing Siglec-7 expression profile and role in fresh breast cancer biopsies from Moroccan patients, along with two other independent cohorts, TCGA and METABRIC. Together, our data strongly suggest that Siglec-7 operates as an immune evasion biomarker, and could be used as a potential target in breast cancer immunotherapy.

## 2 Materials and methods

### 2.1 Patients and samples

In this study, mRNA expression was assessed in 45 breast cancer patients diagnosed with invasive breast carcinoma and treated at the Mohamed VI Oncology Center, Ibn Rochd University Hospital Center, between 2019 and 2023. A total of 90 freshly resected specimens were collected immediately post-surgery, comprising tumor tissues (n = 45) and paired adjacent non-invaded tissues (n = 45), which served as controls. Eligible patients met specific inclusion criteria, notably a confirmed diagnosis of invasive breast carcinoma, complete documentation, as well as free and informed consent. Exclusion criteria included male patients, lack of informed consent, absence of matched control tissues, non-detection of β-actin housekeeping gene expression, and incomplete medical records. Clinicopathological data, such as age, grade, molecular subtypes, and biomarker status (ER, PR, HER2, and Ki-67), were assigned according to standard protocols and guidelines and sourced from hospital records.

### 2.2 Transcriptomic datasets acquisition and preprocessing

Complementing our in-house cohort analysis, the computational study integrated transcriptomic and clinicopathological data from primary invasive breast carcinoma patients, sourced from two major datasets, The Cancer Genome Atlas (TCGA) American cohort and the Molecular Taxonomy of Breast Cancer International Consortium (METABRIC) Briton-Canadian cohort. Data were retrieved from the open-access cancer genomics interface, cBioPortal (https://www.cbioportal.org/), and subsequently, clinicopathological parameters from patient and sample files were integrated and mapped to the corresponding gene expression data, including RNA-seq for TCGA and microarray data for METABRIC. The analysis included only patients with complete transcriptomic and clinicopathological profiles, excluding male patients and those with incomplete records, resulting in a final cohort of 1070 patients from TCGA and 1980 patients from METABRIC. In addition, for the TCGA clinicopathological assessment, PAM50 molecular subtyping and ER, PR, and HER2 status were sourced from The Cancer Immunome Atlas (TCIA) (34) platform (https://tcia.at/home) and meticulously matched to patient IDs. For survival prediction in METABRIC, the Nottingham Prognostic Index (NPI) score was employed to stratify patients into four distinct prognostic categories. During the preprocessing phase of our analysis, raw gene expression counts from RNA-seq data were log-transformed as needed, while the microarray data were originally provided in log2-transformed and normalized formats. To ensure result reliability, all analyses and statistical tests were independently repeated by two investigators.

### 2.3 Total RNA isolation and reverse transcription

The isolation of total RNA from 90 fresh biopsy samples, comprising 45 breast carcinoma tissues and 45 paired adjacent non-invaded control tissues, was performed using TRIzol reagent (Invitrogen, France), according to the manufacturer’s protocol. The total RNA, resuspended in RNase-free water, was either immediately used for subsequent steps or preserved at -80°C for long-term storage. The concentration and quality of RNA were assessed using a NanoVue™ Plus spectrophotometer (GE Healthcare, UK). To obtain the complementary DNA (cDNA), reverse transcription was performed using 0.5 μg of total RNA, ensuring consistency across sample comparisons. The reaction mixture was prepared with 1 µL of Random Hexamer Primer (25 µg, Bioline, France), 0.5 µL of RNase inhibitor (Invitrogen, France), 0.5 µL of Tetro Reverse Transcriptase enzyme (Bioline, France), 4 µL of Tetro Reverse Transcriptase buffer, and 4 µL of dNTPs (10 mM). RNase-free water was added to adjust the final volume to 20 µL. The reaction mixture was subjected to the following incubation program: 25°C for 10 minutes, 42°C for 45 minutes, 70°C for 15 minutes, and held at 4°C.

### 2.4 Real-time PCR assay

Gene expression was relatively quantified using real-time PCR with the fluorescent dye SYBR™ Green PCR Master Mix (Thermo Fisher) on the croBEE^®^ and Bio-Rad CFX96 real-time PCR detection systems. Specific primer pairs for A2AR, VISTA, and the internal reference gene β-Actin were used at 10 μM, while for Siglec-7, a concentration of 25 μM was applied. A reaction mixture was prepared, consisting of 10 µL SYBR™ Green, 7 µL H_2_O, and 0.5 µL of each forward and reverse primers, resulting in an 18 µL mixture per reaction. To each well of the PCR microplate, 2 µL of cDNA was added, and for the negative control, 2 µL of ultra-pure water was used instead. The PCR cycling program was designed with an initial 10-minute holding stage at 95°C to activate the polymerase and denature the samples, followed by 40 cycles consisting of 15 seconds of denaturation at 95°C and 1 minute of annealing and extension at 60°C. Post-amplification, specificity was confirmed through analysis of the amplicon melting curves. If deemed necessary, a second verification step involved performing agarose gel electrophoresis of the PCR products to ensure the integrity and specificity of the amplified cDNA. The Ct values were determined from fluorescence data collected at the end of each PCR cycle’s extension phase. Following the application of the 2_-ΔCt_ method for tumor and paired control tissue comparison, relative quantification was computed using the approach 2_-ΔΔCt_ approach. The following primer pairs were employed in this study (**Table 1**):

**Table 1:** Primer sequences used for real-time PCR.

Genes	Forward Sequences	Reverse Sequences
β-actin	5′-GAGATGGCCACGGCTGCTT-3′	5′-GCCACAGGACTCCATGCCCA-3′
SIGLEC-7	5′-AAGAAGCCACCAACAATGAG -3′	5′-CAGTTAGACAAGAGGAATAAGTTC -3′
A2AR	5’ATCGCCATTGACCGCTACAT3-’	5’-GCTGACCGCAGTTGTTCCA-3’
VISTA	5TGTAGACCAGGAGCAGGATG-3′	5-ATGCACCATCCAACTGTGTG-3′

### 2.5 Differential gene expression analysis

To uncover genes with significant changes in relation to Siglec-7 expression, a differential gene expression analysis was conducted. Gene expression levels were compared between Siglec-7-High and Siglec-7-Low groups, determined based on the median gene expression value. The High group served as the reference, with the Low group used as the control. For the TCGA RNA-Seq data, differential gene expression analysis was conducted using DESeq2 package (v1.46.0) (35), whereas for the METABRIC microarray data, limma package (v3.36.2) (36) was employed in R-Studio (v2024.04.0 + 735). TCGA normal count data were pre-filtered to exclude rows with low gene counts, while METABRIC data, being pre-normalized, did not require this step. Results were processed using an adjusted p-value threshold of 0.05 (Benjamini-Hochberg correction) and log2 fold-change thresholds of ±1 for TCGA and ±0.01 for METABRIC datasets. Volcano plots were generated to visualize the top upregulated and downregulated genes.

### 2.6 Gene set enrichment analysis

Complementary to the DGE analysis, changes in predefined gene sets related to different biological pathways between High and Low Siglec-7 phenotypes were investigated through pathway-based functional exploration using Gene Set Enrichment Analysis (GSEA) software (v4.3.2) (37). Pathways from the Hallmark, Curated, and Gene Ontology (GO) databases were assessed and reorganized into categories pertinent to the study’s hypotheses. Gene sets were retrieved via the GSEA interface (https://www.gsea-msigdb.org/gsea/downloads.jsp), and analysis was performed on log-normalized expression data, employing 1,000 permutations to estimate nominal p-values, with significance thresholds set at p < 0.05 and FDR < 0.25.

### 2.7 Estimation of tumor-infiltrating immune cell abundance

First, we utilized the ImmuCellAI R package (v2022) (38) to estimate the abundance of infiltrating cells within breast TME. The RNA-Seq module was employed to analyze raw gene expression data from TCGA, whereas the microarray module was used to process the log-normalized data from METABRIC. Subsequently, the deconvo_tme method from the IOBR package (version 1.0) (39) was applied to assess Cibersort abundance scores of NK cell phenotypes (resting and activated) in the TCGA RNA-Seq dataset. Finally, using the z-score method from the same package, we derived signatures for additional immune cell types, including Tumor-Associated Macrophages (TAM), Cancer-Associated Fibroblasts (CAF), and Myeloid-Derived Suppressor Cells (MDSC). The resulting scores were categorized into SIGLEC-7 low and SIGLEC-7 high groups as previously defined.

### 2.8 Digital cytometry-based gene expression profiling in immune cells

To profile the expression of immune checkpoints at the intracellular level within immune cells, the CIBERSORTx digital cytometry approach was employed (40). Access to the CIBERSORT.R source code was granted through a token provided by the corresponding team. Subsequently, pseudo-bulk data for NK, CD8, and CD4 immune cells were estimated from bulk data within a Docker environment, implemented through Docker Desktop software (v4.24.0), utilizing the LM6 immune cell signature matrix (41). To meet the requirement for non-log linear expression data, METABRIC microarray expression data were transformed from log2 scale back to linear scale by computing 2 raised to the power of each log2 intensity value using R-Studio.

### 2.9 Immune-related scores analysis

To evaluate various mechanisms of immune suppression across our groups of interest, we applied three distinct immune-related scoring algorithms: ESTIMATE, gene signature z-scoring, and Tumor Immune Dysfunction and Exclusion (TIDE) analysis. First, the ESTIMATE algorithm was used to calculate immune and tumor purity scores. Subsequently, we employed gene signature z-scoring to evaluate T-cell exhaustion, senescence, anergy, and evasion mechanisms. Both analyses were conducted using the IOBR package via R-Studio. Additionally, the TIDE module (42) was accessed through its official interface (http://tide.dfci.harvard.edu/) to derive tumor T-cell exclusion and T-cell dysfunction scores. Prior to the TIDE analysis, data normalization was performed in R-Studio, in line with the algorithm’s guidelines, by subtracting the row means (average expression of each gene across all samples) from the original expression values, thus centering the data.

### 2.10 Gene expression and breast cancer therapy response dataset analysis

To assess the relationship between Siglec-7 expression and resistance to conventional breast cancer therapies, we analyzed data from two additional cohorts: the Pathological Complete Response (n=1775) and Relapse-Free Survival at 5 years (n=1329) (43). These datasets were obtained from the ROC Plotter platform (https://rocplot.org/). We specifically downloaded Siglec-7 expression data for patients treated with endocrine therapy, anti-HER2 therapy, and chemotherapy, categorizing the expression based on patients’ responder or non-responder status. Siglec-7 mRNA expression was normalized using log2 transformation, and a heatmap was generated to visualize expression differences between responders and non-responders for each of the therapies mentioned above.

### 2.11 Immunophenoscore, Carcinoma EcoTyper, and immunotherapy response analysis

Immunotherapy response in relation to Siglec-7 expression status was investigated using documented parameters, namely Immunophenoscore (34) and Carcinoma EcoTyper (44) analyses. Immunophenoscores were computed for all patients using IPS (v1.0) R package and then compared between Siglec-7 low and high groups. For visualization, gene expressions were averaged within each group, and the resulting data were displayed in an Immunophenogram generated with ggplot2, grid, and gridExtra in R-Studio. For the Carcinoma EcoTyper analysis, transcriptomic data from both Siglec-7 low and high groups were analyzed via the official interface (https://ecotyper.stanford.edu/).

### 2.12 Statistical analysis

All data visualization and statistical computations were conducted using GraphPad Prism (v10.2.3) and R-Studio (v2024.04.0 + 735). Shapiro-Wilk and Kolmogorov-Smirnov normality tests were employed to evaluate the distribution of the data. Gene expression differences between breast cancer tumor tissues and matched adjacent uninvaded tissues were analyzed using the paired Wilcoxon Signed Rank test. Mann-Whitney test was utilized for assessing differences between two independent groups, while the Kruskal-Wallis test was applied for comparing gene expression across multiple groups. For unpaired grouped data, Welch t-test was applied to compare mean expression, accounting for the standard deviations and sample sizes of each group. Spearman’s non-parametric test was used to estimate the correlation coefficients and corresponding p-values between two variables. The prognostic significance of Siglec-7 was evaluated through Kaplan-Meier survival analysis, with overall survival outcomes compared using the log-rank (Mantel-Cox) test. The threshold for statistical significance was set at a two-sided p-value of less than 0.05 for all tests.

### 2.13 Study approval and ethical consent

Written informed consent was obtained from all participants, and for those in critical circumstances, from family members or legal guardians. Prior to giving consent, participants or their legal representatives were fully briefed on the study’s aims and objectives. The study strictly adhered to the ethical standards outlined in the Helsinki Declaration and was approved by the Ethics Committee for Biomedical Research (CERB) of Ibn Rochd (approval code: 28/15).

## 3 Results

### 3.1 Elevated Siglec-7 expression correlated with tumor malignancy and unfavorable clinicopathological outcomes in patients with invasive breast carcinoma

**Figure 1:**
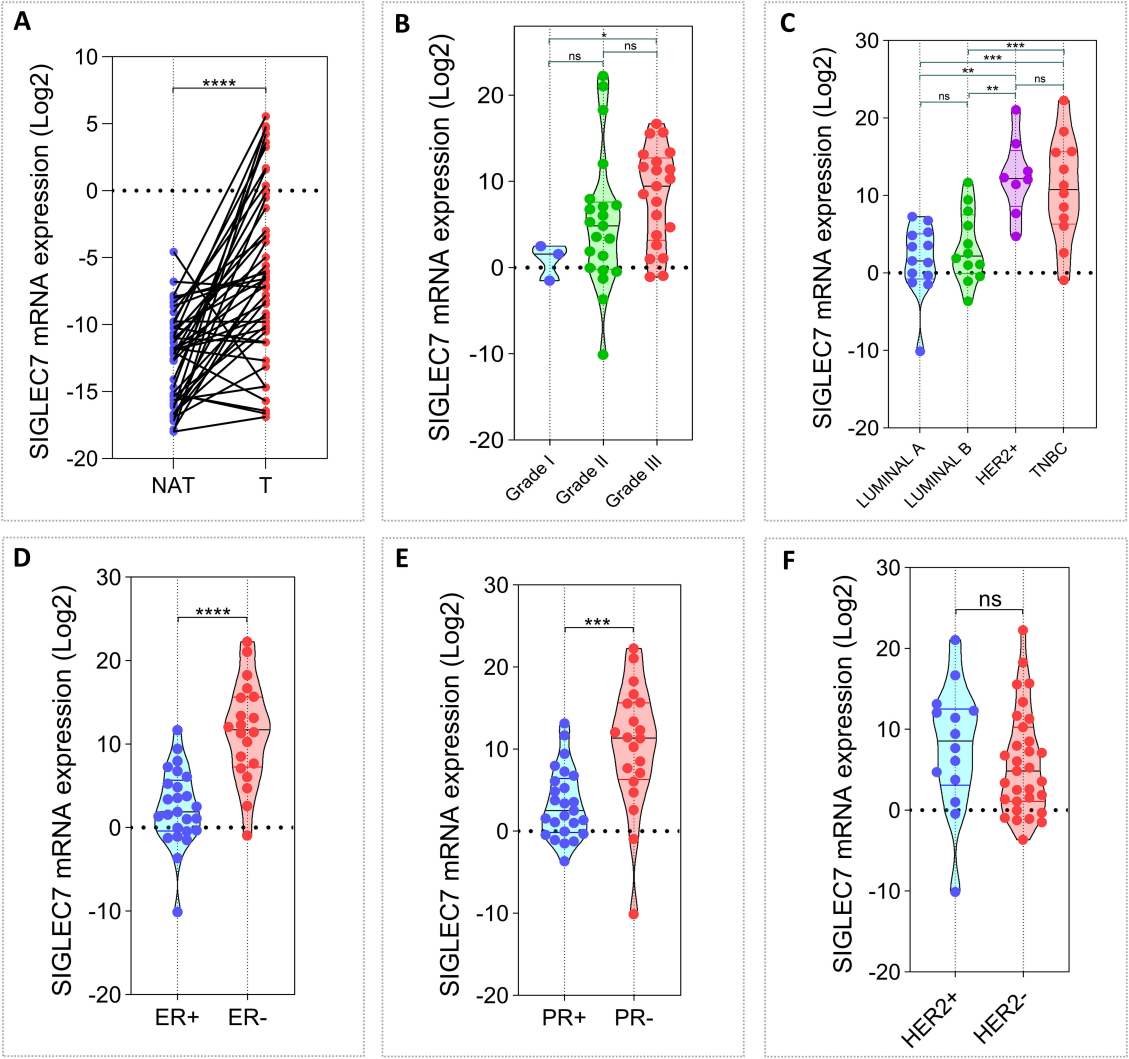
Associations between Siglec-7 gene expression, breast malignancy, and clinicopathological features in our in-house breast cancer cohort (n= 45). Siglec-7 transcript levels were measured using RT-PCR, with expression normalized to β-actin and analyzed relative to matched control tissues, presented as mRNA expression log^2^(^2^(–ΔΔCt)). **(A)** Breast tumor tissues (T) exhibit significantly higher Siglec-7 mRNA levels compared to non-invaded matched adjacent tissues (NAT). **(B)** Siglec-7 gene expression is upregulated in grade III breast cancer compared to grade I (p=0.0405). **(C)** Siglec-7 gene expression is strongly elevated in HER-enriched and TNBC compared to luminal subtypes (HER2+ vs LumA: p = 0.0001, HER2+ vs. LumB: p = 0.0011, TNBC vs. LumA: p = 0.0008, TNBC vs. LumB: p = 0.0068). **(D, E)** Siglec-7 transcript levels are markedly higher in tumors with negative ER and PR status compared to positive phenotypes (ER+ vs. ER-: p < 0.0001, PR+ vs. PR-: p = 0.0002). **(F)** Siglec-7 expression is not significantly associated with HER2 status (p = 0.2375). Statistical significance was established at p < 0.05, with **p < 0.01, ***p < 0.001, ****p < 0.0001, and ‘ns’ denoting no statistical significance.

To explore the expression pattern of Siglec-7 and assess its potential role in human breast tumorigenesis, we initially analyzed an in-house cohort of 45 invasive breast carcinoma patients (**Supplementary Table 1**). Siglec-7 mRNA levels were measured by RT-PCR in 90 fresh tissue samples, evenly divided between breast tumor samples (T) and their corresponding non-invaded adjacent tissues (NAT). Expression levels were normalized to β-actin. First, we observed that breast tumor tissues exhibited significantly higher levels of Siglec-7 transcripts compared to their matched controls (p < 0.0001). Building on this finding, we sought to investigate the clinical relevance of Siglec-7 in our cohort, examining its association with patient age, tumor grade, molecular subtypes, relevant biomarkers, and TNM classification. Initially, we observed that Siglec-7 gene expression was significantly upregulated in grade III breast cancer tissues compared to grade I tumors (p = 0.0405) (**Figure 1B**). Moreover, Siglec-7 gene transcripts were notably elevated in HER2-enriched and triple-negative breast cancers compared to luminal A and B subtypes (**Figure 1C**). Interestingly, Siglec-7 mRNA expression was linked to negative estrogen receptor (ER) and progesterone receptor (PR) status, while no significant correlation was observed with HER2 status (**Figures 1D–F**). Regarding patient age, Ki-67 status, and TNM classification, no significant association was observed with Siglec-7 transcript levels (**Supplementary Figures 1G–K**). These results suggest that higher levels of Siglec-7 expression were associated with more aggressive tumor behavior and unfavorable clinicopathological characteristics.

**Figure 2:**
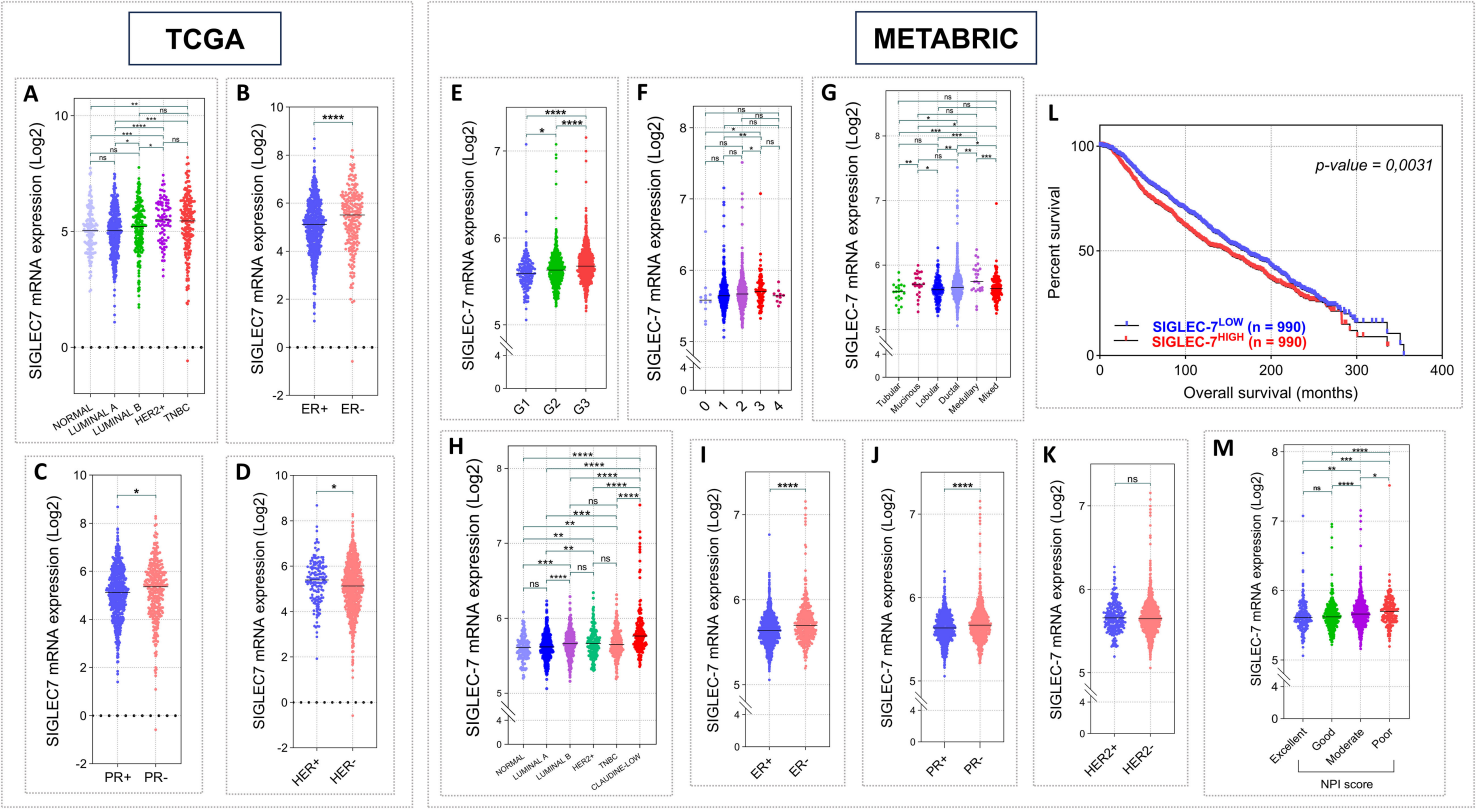
Elevated Siglec-7 transcript levels correlated with unfavorable clinicopathological outcomes in patients with invasive breast carcinoma, based on RNA-seq and microarray data from the TCGA (n = 1070) and METABRIC (n = 1980) cohorts. Log2 normalization was applied to the raw data counts from TCGA for visualization. **(A, H)** Siglec-7 mRNA relative expression is significantly increased in aggressive breast cancer PAM50 molecular profiles, notably TNBC within TCGA cohort and Claudin-low within METABRIC, compared to subtypes linked to favorable outcomes. **(B, C, I, J)** Siglec-7 gene shows a strong association with negative phenotypes of PR and ER biomarkers in both TCGA and METABRIC cohorts. **(D, K)** HER2 status showed no association with Siglec-7 transcripts in METABRIC dataset, but significant differences were observed in TCGA cohort (HER2+ vs. HER2-: p = 0.0119). **(E, F)** Breast tumors with advanced grade and higher stage exhibited a significant increase in Siglec-7 expression compared to those with less severe tumor profiles. **(G)** Aggressive ductal histological type exhibit markedly higher Siglec-7 transcript levels compared to lobular (p=0.0041). **(L, M)** Patients presenting poor prognostic index demonstrate significantly higher Siglec-7 expression compared to those with excellent (p = 0.0001), good (p < 0.0001), or moderate (p = 0.0298) scores. Kaplan–Meier analysis indicated that high Siglec-7 expression is linked to significantly diminished survival outcomes in breast cancer patients. *p < 0.01, **p < 0.01, ***p < 0.001, ****p < 0.0001, and ‘ns’ indicates no significance.

To corroborate our initial findings, we expanded the clinical value analysis of Siglec-7 to encompass 1070 patients from TCGA and 1980 from METABRIC cohorts (**Supplementary Table 2**, **Supplementary Table 3**). Siglec-7 mRNA levels were substantially higher in aggressive molecular subtypes of breast cancer, with particularly strong increases in HER2+ and TNBC subtypes in TCGA cohort, and the Claudin-low subtype in METABRIC patients (**Figures 2A, H**). Consistently, Siglec-7 expression was strongly associated with negative ER and PR phenotypes. Although no correlation was observed between HER2 status and Siglec-7 expression in METABRIC patients, a significant association was identified in TCGA cohort (p = 0.0119) (**Figures 2D, K**). Furthermore, higher tumor grades and stages were significantly associated with increased Siglec-7 expression (**Figures 2E, F**). Ductal carcinoma histological type showed significantly higher Siglec-7 mRNA than lobular (p=0.0041) (**Figure 2G**). Strikingly, poor Nottingham Prognostic Index (NPI) categories were linked to increased Siglec-7 expression, which was further corroborated by Kaplan-Meier survival analysis, showing that high Siglec-7 levels are a significant predictor of poor survival outcomes in breast cancer patients. Contrary to the previous results, TCGA analysis did not reveal a significant association between Siglec-7 gene expression and tumor stage, histological type, TNM classification, or patient survival outcomes (**Supplementary Figure 2**).

In light of the preceding findings, our results underscore the prognostic significance of Siglec-7 expression, as it predicts adverse clinical features and negatively impacts the overall survival of breast cancer patients.

### 3.2 Patients exhibiting high levels of Siglec-7 mRNA showed a significant association with signaling pathways driving breast cancer pathogenesis and progression

**Figure 3:**
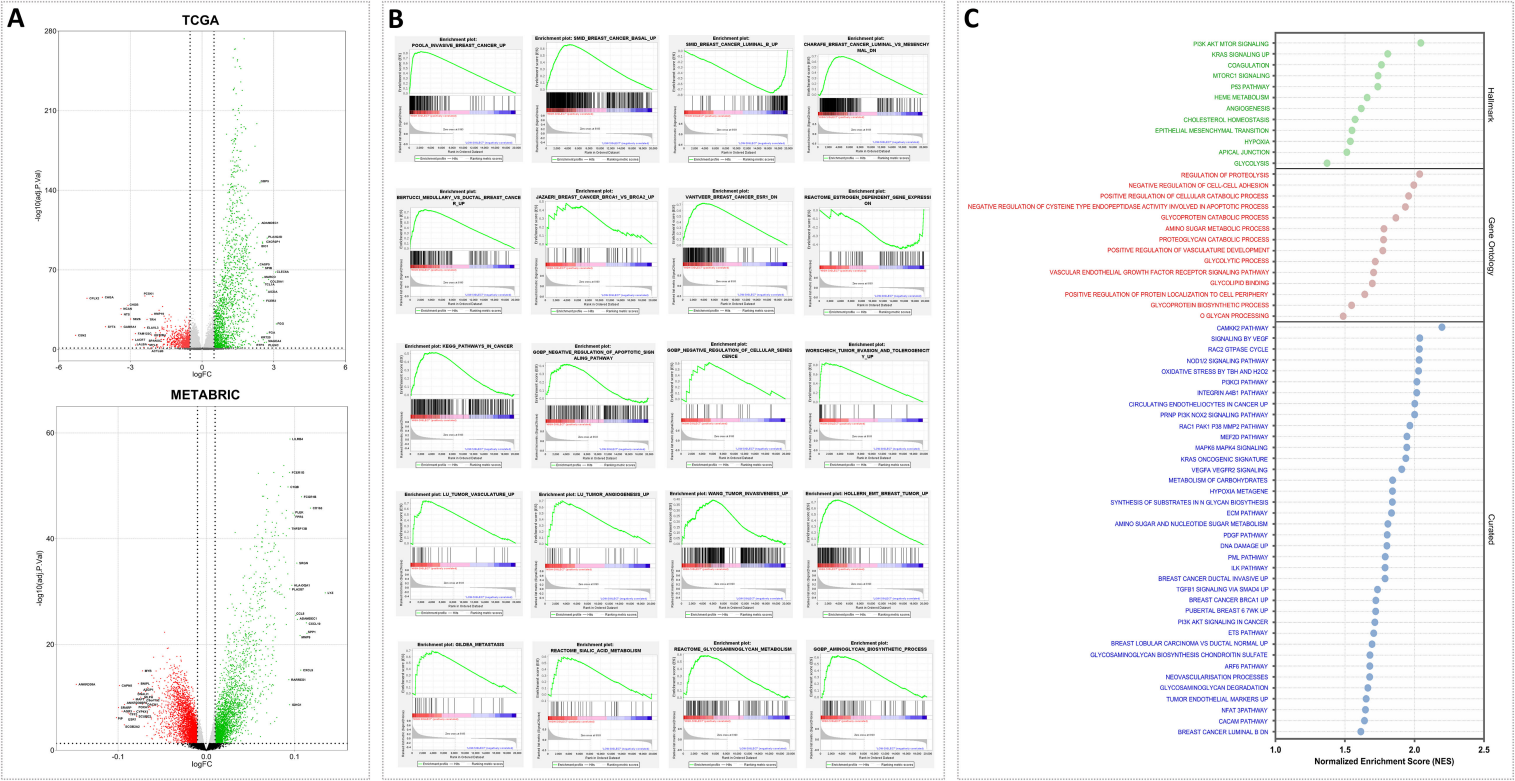
Siglec-7 was linked to gene expression variations and was associated with signaling pathways and biological functions involved in breast cancer pathogenesis, as identified through differential gene and Gene Set Enrichment Analysis. **(A)** Volcano plot illustrating differentially expressed genes that are upregulated (green) or downregulated (red) in the Siglec-7^HIGH^ group across the TCGA and METABRIC datasets. The top 20 genes are labeled. **(B)** GSEA plots demonstrate statistically significant differences in predefined gene sets between Siglec-7^LOW^ and Siglec-7^HIGH^ groups. The plots identify pathways critical for breast cancer development, progression, and aggressiveness, with positive or negative enrichment observed in Siglec-7^HIGH^ TCGA patients. **(C)** Additional key biological processes associated with cancer proliferation, invasion, angiogenesis, and metastasis in TCGA Siglec-7^HIGH^ cluster are depicted in the bubble plot, utilizing molecular signatures from Hallmark, Ontology, and Curated databases. Terms with a nominal p-value < 0.05 and a false discovery rate (FDR) < 0.25 are considered statistically significant and represented. NES stands for Normalized Enrichment Score, and GO refers to Gene Ontology.

Considering the elevated levels of Siglec-7 in mammary tumors, we hypothesized that its gene expression may correlate with alterations in molecular pathways driving breast cancer pathogenesis. For this purpose, differential gene expression (DGE) and gene set enrichment analysis (GSEA) were carried out to uncover the biological processes and mechanisms implicated in cancer development and progression. Patients were organized into two clusters, Siglec-7^low^ and Siglec-7^high^, using the median expression value as the cutoff. Of note, the volcano plots (**Figure 3A**) revealed that Siglec-7 expression was associated with significant gene regulation changes, with the top 20 upregulated and downregulated genes marked. These findings suggested that Siglec-7 may influence various biological pathways, as evidenced by the distinct gene expression profiles between Siglec-7^low^ and Siglec-7^high^ clusters. To further investigate, we analyzed predefined gene sets from three human molecular signature databases (Hallmark, Curated, and Ontology) linked to key biological processes involved in cancer proliferation, invasion, angiogenesis, and metastasis. Remarkably, Siglec-7^high^ phenotype was significantly enriched with pathways linked to oncogenesis and breast cancer progression. Indeed, Siglec-7 correlated with signatures of invasive basal breast cancer and more aggressive phenotypes, encompassing major cancer hallmarks, as illustrated in (**Figures 3B, C**). More critically, our analysis revealed that gene sets associated with Siglec-7 potential ligands, specifically those involved in sialic acid and aminoglycan metabolism, were significantly enriched. This observation strongly suggests that Siglec-7 might play a pivotal role in breast cancer progression and its aggressive behavior, primarily through immunological mechanisms. The same pathways were analyzed in the METABRIC dataset, showing functionally similar but not identical patterns (**Supplementary Figure 3**).

### 3.3 Siglec-7^high^ breast tumors were associated with an immune-enriched tumor microenvironment

**Figure 4:**
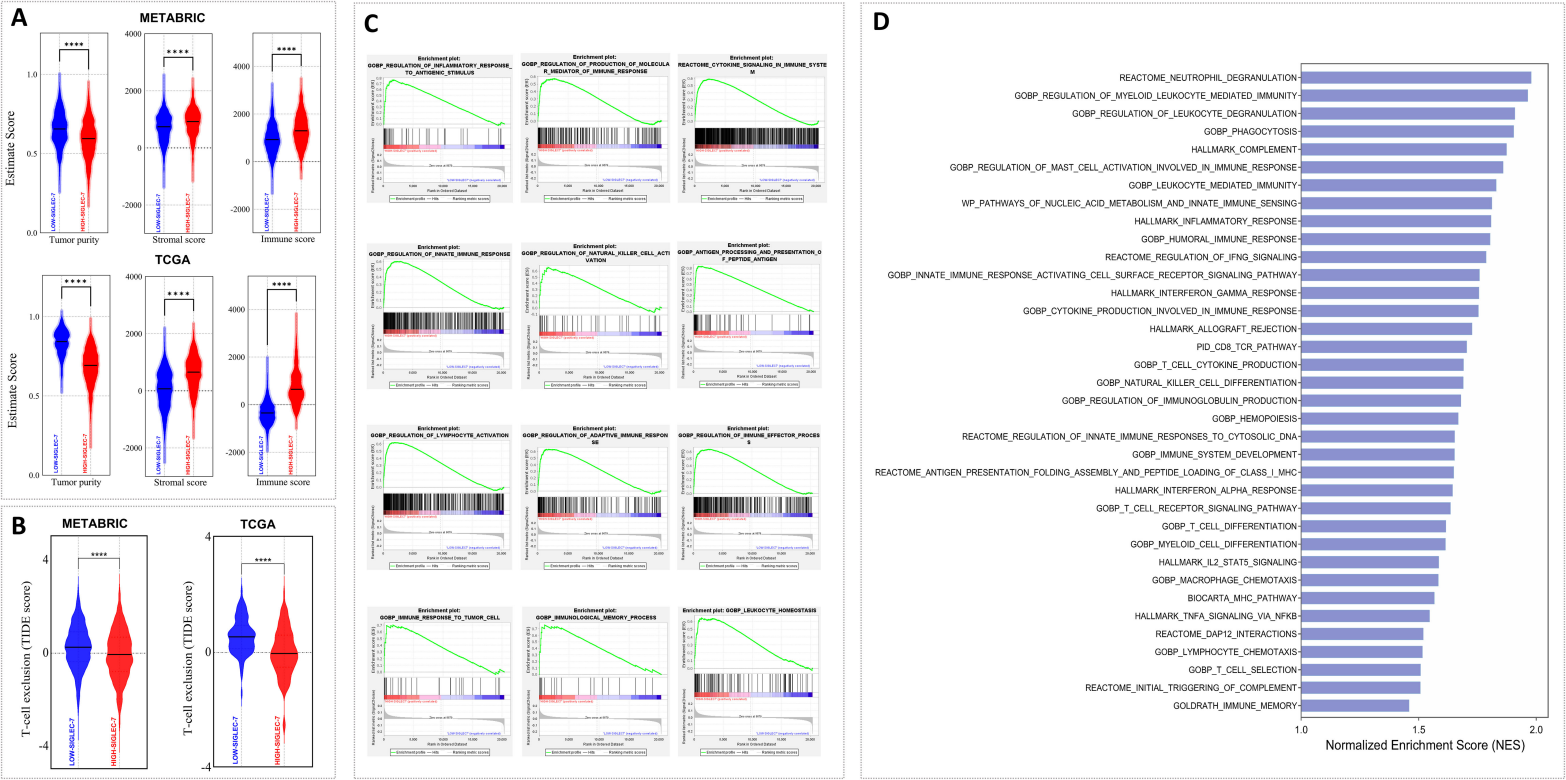
Patients with elevated Siglec-7 expression exhibited a more immune-infiltrated tumor microenvironment and a positive enrichment of major immune function pathways. Estimate, TIDE, and GSEA analyses were used to assess the computed scores. **(A, B)** Siglec-7 expression correlates with a more complex breast tumor microenvironment, marked by increased cellular diversity with elevated immune and stromal scores, in addition to decreased T-cell exclusion in both TCGA and METABRIC patients. **(C, D)** GSEA and bar plots depicting significant positive enrichment of key immune biological processes in METABRIC Siglec-7 high patients. Statistical significance was determined at p < 0.05 and FDR < 0.25, with **** indicating p < 0.0001.

Given the aggressive behavior of Siglec-7^high^ breast tumors, assessing their immune microenvironment dynamics became of particular interest. Consequently, tumor purity, T-cell exclusion potential, and key immune mechanisms were evaluated using Estimate, TIDE, and GSEA analyses. In fact, Siglec-7 expression was linked with an immune-enriched, complex breast tumor microenvironment across TCGA and METABRIC cohorts. This association was characterized by a more heterogeneous tumor composition, with elevated immune and stromal scores, and a notable reduction in tumor T-cell exclusion potential (**Figures 4A, B**). More importantly, functional pathway analysis demonstrated significant positive enrichment of key immunoregulatory biological processes involved in both innate and adaptive responses in patients with high Siglec-7 expression (**Figures 4C, D**; **Supplementary Figures 4E, F**), indicating an active role for Siglec-7 in modulating the immune landscape of breast tumors.

### 3.4 Patients with Siglec-7^high^ expression demonstrated abundant protumoral cell infiltration

**Figure 5:**
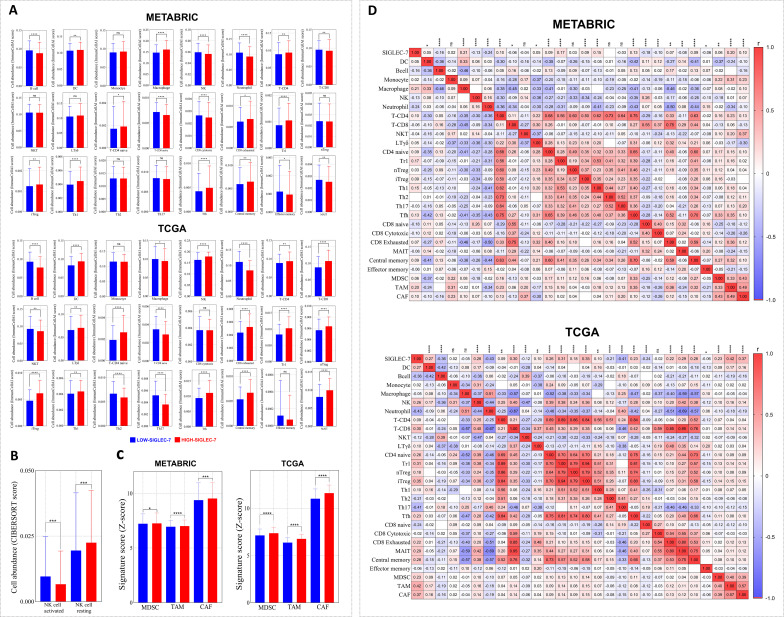
Immune cell infiltration patterns in Siglec-7^high^ breast tumors reflected an immunosuppressive microenvironment. Immune cell fraction distributions were evaluated using the ImmunocellAI algorithm, with complementary analyses performed using Cibersort and Z-score signatures. **(A)** Immune cell distributions analyzed using ImmunocellAI in METABRIC and TCGA cohorts. **(B)** NK cell phenotype infiltration assessed with CIBERSORT in the TCGA dataset. **(C)** Profiles of immunosuppressive cells based on Z-score analysis. **(D)** Siglec-7 expression and its correlation with immune cell fractions in both METABRIC and TCGA cohorts. For statistical comparisons, Mann-Whitney U test was employed to determine significance between Siglec-7 low and high groups, while Spearman correlation coefficient was computed to evaluate associations. *p < 0.05, **p < 0.01, ***p < 0.001, ****p < 0.0001, and ‘ns’ indicates no significant difference.

Building on our previous findings of enriched immune pathways in breast cancer patients with high Siglec-7 expression, and recognizing that immune cell infiltration is a key orchestrator of cancer progression, we hypothesized that Siglec-7 could be a key modulator of immune cell abundance in breast cancer. The Immune Cell Abundance Identifier (ImmuCellAI) algorithm was primarily used to estimate the abundance of 24 immune cell types, with additional analyses conducted using Cibersort and Z-score signatures. In METABRIC cohort, patients exhibiting elevated Siglec-7 expression demonstrated a significant reduction in the infiltration of key effector cells, including B cells, NK cells, mucosal-associated invariant T (MAIT) cells, effector memory T cells, and strikingly, CD8+ cytotoxic T cells. In contrast, there was a marked increase in CD8+ exhausted T cells, accompanied by a pronounced infiltration of immunosuppressive regulatory T cells, specifically Tr1 and induced Treg (iTreg) cells (**Figure 5A**). Furthermore, elevated Siglec-7 expression was positively linked with the presence of myeloid-derived suppressor cells (MDSCs), tumor-associated macrophages (TAMs), and cancer-associated fibroblasts (CAFs), reinforcing a shift toward a more immunosuppressive tumor microenvironment (**Figure 5C**). In TCGA patients, similar patterns of immunosuppression with distinct characteristics were observed. Tumors with high Siglec-7 expression displayed abundant infiltration of protumoral cells, including various Treg subpopulations (Tr1, nTreg, iTreg), MDSCs, TAMs, and CAFs (**Figures 5A, C**). Conversely, an increased presence of NK cells and CD8+ T cells was observed. Notably, among CD8+ phenotypes, there was a significant infiltration of exhausted CD8+ T cells in the high Siglec-7 group (p < 0.0001), while no significant differences were found in the cytotoxic phenotype (p = 0.7088). To further characterize the NK cell phenotypes within the tumor microenvironment, we employed the CIBERSORT algorithm to estimate the abundance of activated versus resting NK cells. A significant increase in resting NK cells was noted, coupled with a decrease in activated NK cell infiltration in patients with high Siglec-7 expression, thereby reinforcing the protumoral microenvironment and exacerbating immunosuppression. To solidify our previous observations, we conducted a correlation analysis to explore the association between Siglec-7 expression levels and the abundance of infiltrating immune cells. We observed a strong positive correlation with the infiltration of cells favoring tumor growth in the breast cancer microenvironment (**Figure 5D**), highlighting Siglec-7 significant influence in modulating the immune infiltration profile toward an immunosuppressive state.

### 3.5 Siglec-7 expression in breast cancer defines a hallmark of an inhibitory immune checkpoint profile and impaired antitumoral responses

**Figure 6:**
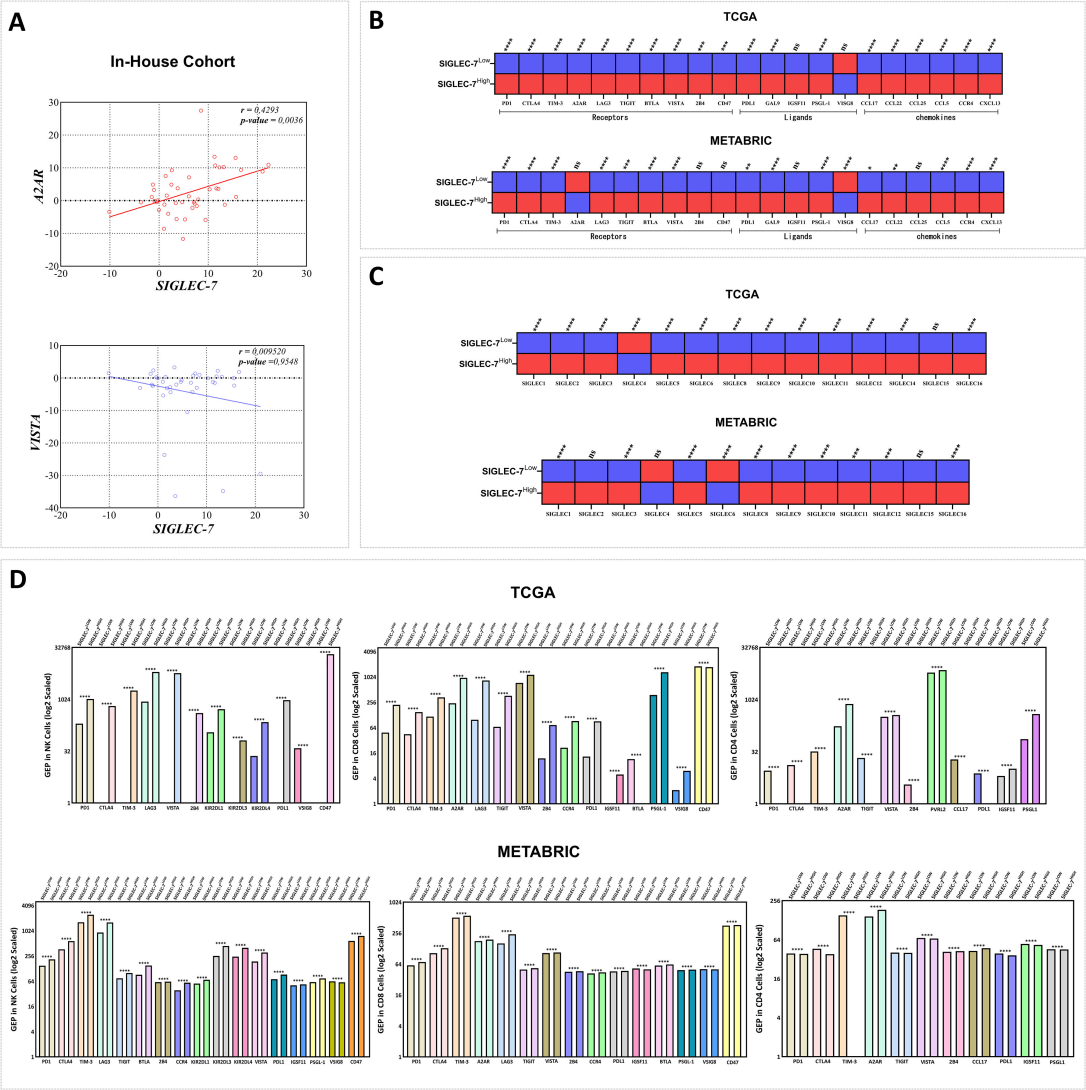
Siglec-7 overexpression correlated with the upregulation of a distinct inhibitory immune checkpoint profile across the tumor microenvironment and immune effector cells in breast cancer patients. **(A)** Siglec-7 expression demonstrates a significant positive correlation with the inhibitory immune checkpoint A2AR (r = 0.4293, p = 0.0036) in our in-house cohort, but not with VISTA (r = 0.09520, p = 0.9548), as assessed using RT-PCR. **(B)** Upregulation of inhibitory immune checkpoints and pro-tumoral molecules is associated with Siglec-7 high expression profile. In the heatmap, red indicates upregulation, while blue represents downregulation. **(C)** Patients with Siglec-7^HIGH^ breast tumors exhibited significant upregulation of several SIGLEC family members, with notable downregulation of SIGLEC4 (p < 0.0001, TCGA) and SIGLEC6 (p < 0.0001, METABRIC). **(D)** Siglec-7 expression is associated with an elevated gene expression profile of inhibitory checkpoints in NK cells, CD8+, and CD4+ T cells, revealing distinct immunoregulatory patterns in breast cancer patients. Intracellular gene expression levels were scaled using the antilog, where 1 denotes the lowest expression. Mann-Whitney U test was used to assess differences between Siglec-7 low and high groups, Spearman correlation coefficient to evaluate associations, and Welch’s t-test to compare mean gene expression profiles across groups, accounting for standard deviations and sample sizes. *p < 0.05, **p < 0.01, ***p < 0.001, ****p < 0.0001, and ‘ns’ for no significant difference.

After delving into the cellular components linked to our gene of interest, we aimed to elucidate the molecular mechanisms defining Siglec-7 distinct immunosuppressive profile, with particular emphasis on inhibitory immune checkpoints and protumoral molecules that orchestrate the immune response within the tumor microenvironment. A2AR and VISTA have emerged as critical immune checkpoints that play a significant role in immune escape within breast cancer, with their upregulation impairing immune surveillance and fostering a microenvironment that supports tumor progression (45, 46). In light of this, we first investigated the relationship between Siglec-7 and these checkpoints in our in-house cohort through RT-PCR analysis (**Figure 6A**). Remarkably, Siglec-7 expression exhibited a significant positive correlation with the inhibitory immune checkpoint A2AR (r = 0.4293, 95% CI [0.1429, 0.6494], p = 0.0036). In contrast, no significant correlation was found with VISTA, which demonstrated a slight, non-significant negative trend (r = 0.0952, 95% CI [-0.3199, 0.3369], p = 0.9548). To gain deeper insight into the molecular immunosuppressive profile of Siglec-7, we expanded our analysis to include a wider panel of inhibitory checkpoints and protumoral molecules associated with breast cancer immune escape, including members of the SIGLEC family as key glycoimmune regulators, within TCGA and METABRIC cohorts. Patients exhibiting high levels of Siglec-7 consistently showed upregulation of multiple inhibitory immune checkpoints, including PD-1, CTLA-4, TIM-3, A2AR, LAG-3, TIGIT, BTLA, VISTA, and their ligands, along with increased expression of pro-tumoral chemokines such as CCL-17, CCL-22, CCL-25, CCL-5, CCR-4, and CXCL-13 (**Figure 6B**). A similar trend was observed in METABRIC patients, although intriguingly, A2AR did not show a significant association with Siglec-7 expression in the bulk tumor samples. These observations were validated through correlation tests in the same cohorts, revealing a strong positive association with the inhibitory molecules (**Supplementary Figure 6E**). Notably, Siglec-7^HIGH^ tumors were also characterized by a marked upregulation of multiple SIGLEC family members — including SIGLEC-1, -2, -3, -5, -6, -8, -9, -10, -11, -12, -14, and -16 — whereas SIGLEC4 was significantly downregulated. A non-significant trend toward SIGLEC15 upregulation was additionally observed. Interestingly, METABRIC patients displayed consistent expression patterns, except for SIGLEC6, which showed a marked downregulation (**Figure 6C**). Correlation analyses conducted in the same cohorts corroborated these findings, reinforcing similar interaction dynamics between Siglec-7 and other SIGLEC family members (**Supplementary Figure 6F**).

Prompted by the results from bulk tumor tissues, and given the potential limitations of bulk expression in fully representing cellular diversity (47), we sought to explore if this upregulation of inhibitory checkpoints and protumoral molecules similarly manifests at the intracellular level in immune effector cells, specifically NK, CD4+, and CD8+ cells. To address this question, we employed intracellular digital cytometry to uncover distinct molecular profiles of immune checkpoints in these effector cells across TCGA and METABRIC cohorts (**Figure 6D**). In TCGA patients, NK cells from tumors expressing high levels of Siglec-7 demonstrated a marked upregulation of inhibitory checkpoints, including PD-1, CTLA-4, TIM-3, LAG-3, PDL1, CD47, and VISTA. Additionally, an array of specific NK inhibitory markers, namely KIR2DL1, KIR2DL3, and KIR2DL4, were elevated, indicative of tumor-induced NK cell suppression. Notably, VSIG8, known as a ligand for VISTA, was downregulated in these high Siglec-7 conditions. Similarly, in METABRIC cohort, NK cells maintained this pattern of upregulated inhibitory checkpoints and exhibited upregulation of additional molecules, particularly TIGIT, BTLA, and IGSF11, with VSIG8 consistently downregulated, reinforcing the hypothesis of Siglec-7 mediated suppression of NK cell activity within breast tumor microenvironment. CD8+ T cells in Siglec-7^high^ TCGA patients displayed a molecular profile similar to that of NK cells, with upregulation of inhibitory checkpoints such as PD-1, CTLA-4, TIM-3, LAG-3, TIGIT, and CCR4, alongside unexpectedly elevated VSIG8 expression and high levels of A2AR. However, CD47 and BTLA were downregulated, suggesting differential modulation of T cell activity in these tumors. In METABRIC patients, this profile was largely consistent, though with upregulation of CD47 and BTLA and a downregulation of VSIG8. These variations emphasize the complexity of Siglec-7 immune modulation of CD8 cells profile within breast tumor microenvironment, potentially involving compensatory mechanisms to fine-tune immune suppression. Distinct patterns of protumoral gene expression were observed in Siglec-7 high CD4+ cells across cohorts. In TCGA patients, the upregulated molecules included VISTA, A2AR, PVRL2, IGSF11, and PSGL-1, while intriguingly, key inhibitory molecules such as PD-1, CTLA-4, TIM-3, TIGIT, 2B4, CCL17, and PD-L1 were downregulated. This trend was more pronounced in Siglec-7 high METABRIC patients, with additional markers including VISTA and IGSF11 showing decreased expression. Notably, upregulation in these patients was largely restricted to A2AR, 2B4, and CCL17, suggesting the potential involvement of alternative pathways in regulating CD4+ T cell function within siglec-7 high breast tumor microenvironment. In view of these results, Siglec-7 appears to play a crucial role in the progression and development of human breast cancer, primarily through the activation of inhibitory pathways in effector cells, particularly those involving A2AR.

**Figure 7:**
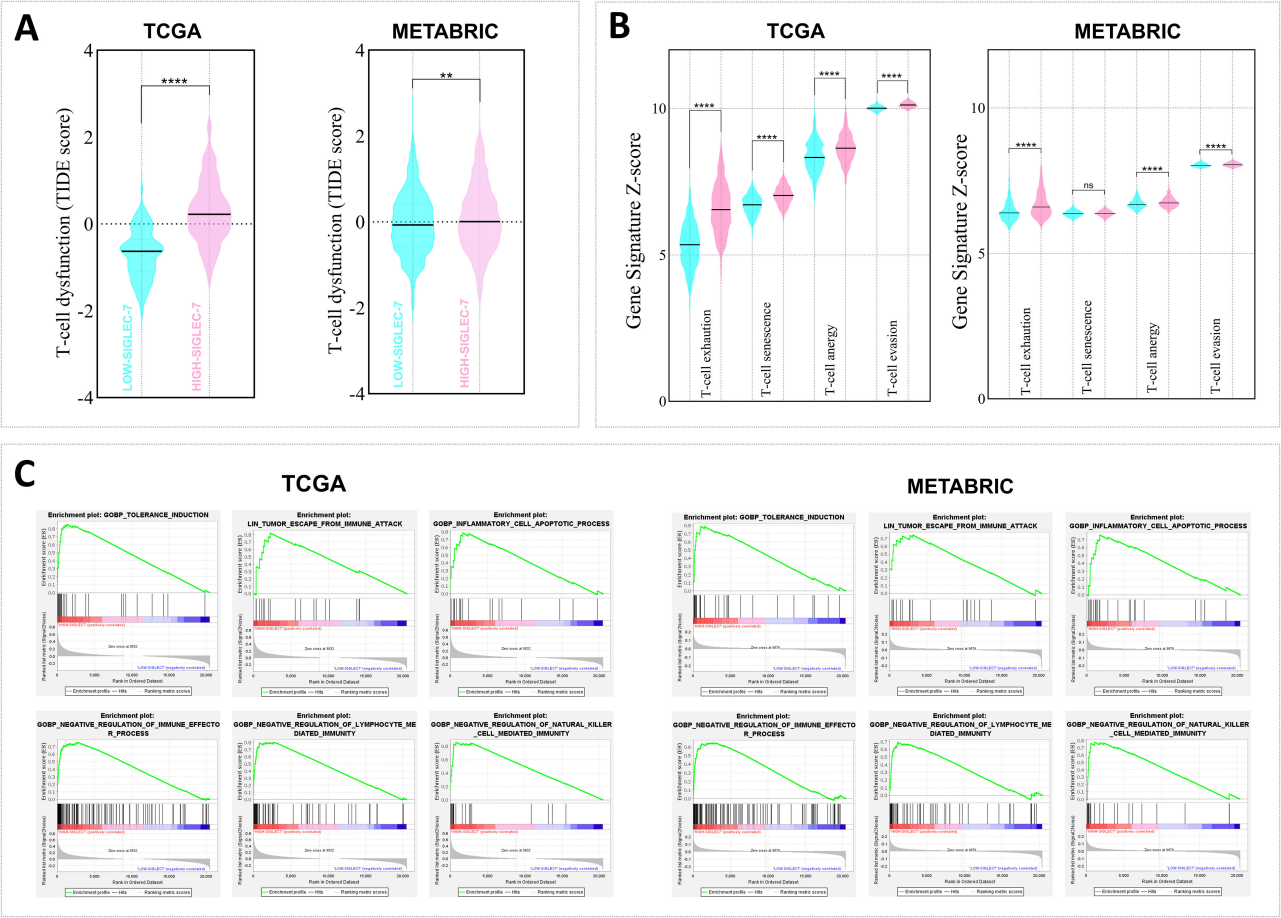
High Siglec-7 expression in breast tumors was strongly linked to impaired antitumoral response signatures. TIDE, Z-score, and GSEA analyses were employed. **(A, B)** Increased Siglec-7 mRNA levels are linked to the upregulation of multiple signatures associated with T-cell dysfunction and immune escape signatures. The cyan color represents Siglec-7^LOW^ group, while the light pink color denotes Siglec-7^HIGH^ group. **(C)** Positive enrichment of significant pathways associated with immune tolerance and downregulation of key cellular effectors was observed in patients with high Siglec-7 expression. Statistical significance was determined at p < 0.05 and FDR < 0.25, with **p < 0.01, ****p < 0.0001 and ‘ns’ for no significant difference.

To further assess the immunosuppressive role of Siglec-7 in breast cancer patients, additional computational analyses were employed to evaluate Siglec-7 gene expression in relation to multiple signatures related to T-cell dysfunction and immune escape (**Figure 7**). Consistently, our findings revealed that patients with elevated Siglec-7 gene expression exhibit a positive association with molecular signatures indicative of extensive T-cell dysfunction (**Figure 7A**). Critically, this pattern is accompanied by the upregulation of various functional impairment phenotypes, including T-cell exhaustion, senescence, anergy, and enhanced T-cell tumor evasion (**Figure 7B**). These associations were further confirmed through correlation analysis, highlighting Siglec-7 major role in impairing T-cell functions (**Supplementary Figure 7**). Furthermore, we observed a marked enrichment in pathways related to tolerance induction, negative immune regulation, and the apoptotic processes of inflammatory cells, all of which contribute to a reduced and impaired anti-tumor immune response (**Figure 7C**). These findings furthermore, we observed a marked enrichment in pathways related to tolerance induction, negative immune regulation, and the apoptotic processes of inflammatory cells, all of which contribute to a reduced and impaired anti-tumor immune response.

### 3.6 Elevated Siglec-7 expression in breast cancer was associated with resistance to standard treatments and predicted a poor response to immunotherapy

**Figure 8:**
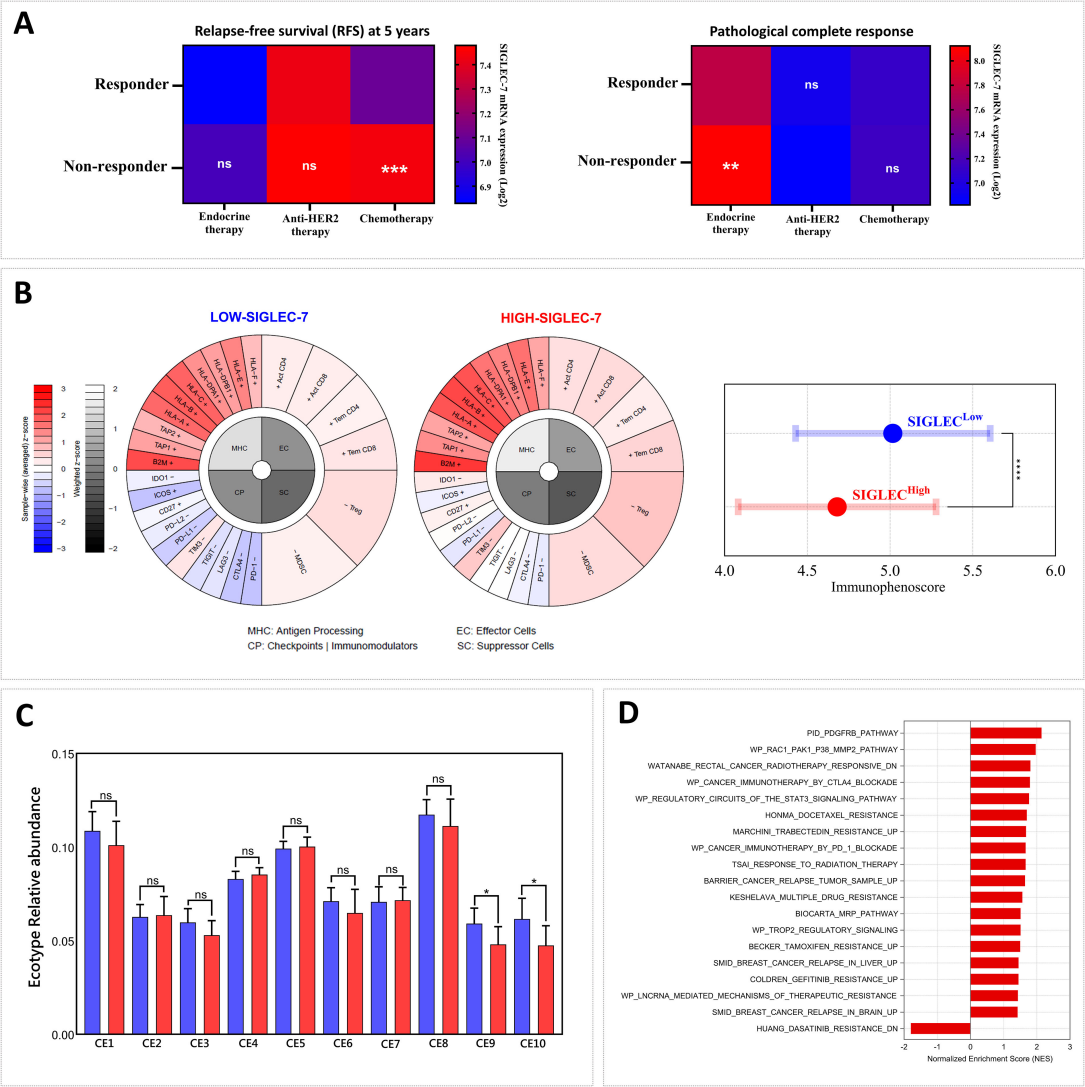
Patients exhibiting high Siglec-7 expression demonstrated resistance to conventional breast cancer therapies and limited response to immunotherapy. **(A)** Non-responders to chemotherapy (p = 0.0001, RFS) and endocrine therapy (p = 0.0066, pCR) show significantly elevated Siglec-7 expression compared to responders. **(B)** High Siglec-7 expression is significantly associated with a lower Immunophenoscore (IPS) and predicts a poorer response to conventional immunotherapy in TCGA patients. The immunophenogram illustrates the IPS parameters between Siglec-7 low and high expression groups. **(C)** Increased Siglec-7 levels are associated with reduced relative abundance of CE9 and CE10 in TCGA cohort, ecotypes linked to favorable trends in immunotherapy response. Red represents Siglec-7^HIGH^ group, while blue indicates Siglec-7^LOW^. **(D)** High Siglec-7 expression in the TCGA cohort correlated with significant enrichment of pathways related to cancer therapy resistance. Significance was established using p < 0.05 and FDR < 0.25, with *p < 0.05, **p < 0.01, ***p < 0.001, ****p < 0.0001 and ‘ns’ for non-significant results.

Breast tumor microenvironment, shaped by its cellular composition and molecular signatures, is integral to determining tumor aggressiveness and profoundly influencing the response to therapeutic interventions, including immunotherapy (11). In light of this, our previous findings linking high Siglec-7 gene expression patterns with a more immunosuppressive and aggressive tumor phenotype led us to question its impact on therapy outcomes. In this regard, we initially examined additional breast cancer therapy response cohorts, focusing on Pathological Complete Response (pCR, n=1775) and 5-year Relapse-Free Survival (RFS, n=1329) to assess the associations between Siglec-7 gene expression and response status to conventional treatments, including endocrine therapy (hormonotherapy), anti-HER2 therapy, and chemotherapy. As expected, significantly elevated Siglec-7 expression was observed in non-responders to chemotherapy (p = 0.0001, RFS) and endocrine therapy (p = 0.0066, pCR), further underscoring its role in predicting poor therapeutic outcomes (**Figure 8A**). Subsequently, we sought to explore the association with response to immune checkpoint inhibitors immunotherapy by evaluating relevant parameters, namely the immunophenoscore and the relative abundance of ecotypes in the TCGA cohort. Our findings revealed that elevated Siglec-7 expression was inversely associated with the immunophenoscore, indicating poorer outcomes with conventional immunotherapy, while patients in the Siglec-7^high^ group also exhibited a decreased relative abundance of CE9 and CE10 ecotypes, further emphasizing its role in unfavorable treatment response (**Figures 8B, C**). In line with our previous findings, the same cohort of patients exhibited a marked enrichment of multiple biological pathways associated with resistance to chemotherapy, radiotherapy, and immunotherapy, as well as pathways implicated in breast cancer recurrence. This consistent pattern suggests a complex interplay of mechanisms through which Siglec-7 may contribute to resistance against various cancer treatments (**Figure 8D**). In METABRIC cohort, similar analyses revealed comparable patterns, though not identical (**Supplementary Figure 8**).

## 4 Discussion

Tumor heterogeneity stands as a hallmark of breast cancer, reflected in the diverse molecular subtypes that play a central role in the variation of clinical outcomes and treatment responses (2). In fact, the growing understanding of the molecular and cellular mechanisms underlying this heterogeneity has redefined breast cancer immunogenicity, highlighting its complex and evolving interplay with the immune system (11). The progression of breast tumors is predominantly facilitated by immune evasion, where inhibitory immune checkpoint molecules serve as crucial mediators of the protumoral effect (48). This strategy has been clinically adopted, as exemplified by Atezolizumab (anti-PD-L1), the exclusive Food and Drug Administration (FDA) approved immunotherapy for breast cancer, though its application remains limited to metastatic triple-negative breast cancer (TNBC) (49). Numerous studies have demonstrated that the synergistic blockade of PD-1/PD-L1 with other inhibitory checkpoints significantly improves survival, underscoring the need to explore additional targets to overcome resistance and expand therapeutic options for all forms of breast cancer (12, 50, 51). To address this, it is crucial to investigate mechanisms of resistance, such as hypersialylation and other compensatory pathways, that may limit the efficacy of current immune checkpoint inhibitors (ICIs) in breast cancer (16, 52, 53). Indeed, dysregulation in sialyltransferase expression leads to an accumulation of sialylated glycans in breast cancer, which, similar to the PD-L1–mediated T cell exhaustion pathway, may promote immune evasion by activating the emerging Siglec immunoreceptors (21). Building on this compelling evidence, the rationale for this study was to investigate Siglec-7 expression, examining its clinical significance, prognostic implications, as well as its potential impact on the immunological landscape and therapeutic outcomes in breast cancer.

Our experimental findings demonstrated that breast tumor tissues exhibit significantly higher levels of Siglec-7 transcript compared to non-invaded matched adjacent tissues. This elevated expression was predominantly observed in high-grade breast tumors and exhibited a strong association with negative ER and PR phenotypes, as well as HER2-enriched and TNBC molecular subtypes. These findings from fresh biopsies suggested that Siglec-7 expression contributes to breast tumor malignancy and is associated with aggressive phenotypic traits. Acknowledging the constraints of our in-house cohort, we expanded our study by incorporating larger datasets, namely TCGA and METABRIC, to further evaluate our hypothesis. Of particular interest, Siglec-7 mRNA was significantly overexpressed in aggressive PAM50 molecular profiles of breast cancer, showing elevated levels in higher-grade tumors, and was particularly upregulated in the Claudin-low subtype of TNBC, known for its extreme aggressiveness (54), further corroborating our experimental findings. Although positive HER2 status was associated with Siglec-7 gene expression in TCGA patients, the gene was predominantly linked to the negative phenotypes of hormone receptors across all cohorts. Of note, no significant association was observed with HER2 status in either METABRIC or our experimental cohort. Importantly, patients with high Siglec-7 expression exhibited a poor prognostic index and were associated with significantly reduced survival outcomes in METABRIC breast cancer cohort, although no such significance was observed in TCGA patients. The observed discrepancies may be attributed to the diverse origins of the cohorts analyzed, including our Moroccan cohort, the American TCGA cohort, and the British-Canadian METABRIC cohort. Despite these differences, it is reasonable to point out that all analyses consistently highlighted a strong association between Siglec-7 expression and aggressive phenotypic traits in breast tumors, making it a potential biomarker worthy of consideration in emerging clinical protocols. Consistently, colorectal cancer patients with a poor prognosis, particularly those with an overall survival (OS) of 2 years or less, displayed significantly higher mean expression levels of Siglec-7 protein (55). Similarly, in prostate cancer, elevated Siglec-7 expression was associated with poorer outcomes (56). In bladder cancer patients, high co-expression of Siglec-6 and Siglec-7 significantly predicted shorter overall and disease-specific survival (57). Siglec-7 also indicated adverse outcomes in ovarian cancer and served as a marker of poor prognosis in glioma (58, 59). In breast tumors, elevated levels of sialic acid were observed in the cancer cell glycocalyx and stroma, particularly within regions of high fibrosis. This increase in sialylation was notably pronounced in HER2+ and Triple Negative tumors, with a marked upregulation of sialoglycoprotein biosynthesis genes, such as ST3GAL4, NANS, and CMAS (21). In line with our results, all these observations support the link between increased Siglec-7 expression and both aggressive clinical outcomes and poor prognosis in cancer. To further assess the hypothesis regarding the association between Siglec-7 and tumor aggressiveness, functional analysis revealed positive enrichment of basal breast cancer pathways, enhanced tumor angiogenesis, invasiveness and hypoxia, as well as pathways related to cancer metastasis. Interestingly, it also highlighted an association with sialic acid metabolism pathways, further supporting the aggressive nature of Siglec-7 high tumors.

To evaluate the immunological significance of Siglec-7 expression in breast cancer, we first examined the immune exclusion potential and the immune pathways involved in tumors with elevated levels of this gene. Fascinatingly, Siglec-7 high breast tumors were associated with an immune-enriched microenvironment, characterized by elevated immune and stromal scores, reduced T-cell exclusion, and substantial enrichment in various pathways regulating innate and adaptive immunity, suggesting Siglec-7 crucial involvement in shaping the immune landscape of breast cancer. In fact, Siglec-7 was documented to be expressed on different cells of the immune system, including tumor infiltrating immune cells (26, 27, 30). Moreover, elevated Siglec-7 expression was linked to aggressive breast cancer subtypes, notably HER2-enriched and Claudin-low TNBC, which are characterized by their high immunogenicity, evidenced by a greater infiltration of tumor-infiltrating lymphocytes (TILs) (11). Siglec-7 high tumors also exhibited reduced purity, a well-established independent factor associated with poor prognosis and aggressive malignancy traits, including immunosuppression (60, 61). In light of these findings, we proceeded to investigate the characteristics and phenotypes of immune infiltrating cells in breast tumors across both Siglec-7 expression groups. Consistent with our hypothesis, patients expressing high levels of Siglec-7 exhibited a strongly immunosuppressive tumor microenvironment, with an overabundance of CD8+ exhausted T cells and a marked presence of immunosuppressive regulatory T cells, including Tr1 and iTreg, as well as MDSCs, TAMs, and CAFs. This environment was marked by a reduced presence of effector cells, particularly CD8+ cytotoxic T cells and activated NK cells, thereby reinforcing the involvement of Siglec-7 in tumor growth in breast cancer patients, essentially through immunosuppressive cell subsets. In pancreatic ductal adenocarcinoma, Siglec-7 on myeloid cells mediates the differentiation of monocytes into macrophages that display immunosuppressive characteristics, triggered by heightened sialylation in tumor cells (56). In fact, antibodies against Siglec-7 and Siglec-9 have demonstrated the ability to inhibit macrophage polarization into TAMs, which in turn reduces the immunosuppressive microenvironment and significantly reduces tumor burden in mouse models (31). Furthermore, MDSCs from glioma patients displayed Siglec-7 expression, suggesting its potential role in MDSC-mediated immunosuppression within the glioma tumor microenvironment (62). In multiple myeloma (MM), *in vitro* functional assays highlighted that interactions between Siglec-7L and Siglec-7R receptors play a crucial role in suppressing NK cell cytotoxicity (63). Notably, it has been reported that Siglec-7 inhibition promotes the activation of both primary T cells and antigen-presenting dendritic cells, highlighting its role in modulating T cell signaling pathways involved in activation and polarization (29). Regarding the high infiltration of Tregs in Siglec-7^high^ patients, it has been documented that the abundance of these cells in breast cancer is also strongly associated with negative hormone receptor status, a positive HER2 profile, lymph node involvement, and notably reduced survival (64–66).

To further investigate the immunosuppressive role of Siglec-7 in breast cancer, we evaluated its correlation with emerging inhibitory checkpoints, specifically A2AR and VISTA, in our in-house cohort. Recent findings have highlighted the significant role of A2AR in the key biological mechanisms driving tumor formation and breast cancer progression, notably by promoting protumoral infiltration and increasing the expression of immunosuppressive molecules (45). Interestingly, we observed a positive correlation between this emerging checkpoint and Siglec-7 gene expression, highlighting a possible synergistic effect between these molecules within the mammary tumor microenvironment. Intriguingly, no significant association was found between VISTA and Siglec-7, although a trend toward a negative correlation was observed. Notably, while VISTA did not correlate with the aggressive characteristics of breast cancer at the transcriptomic level, a clear connection emerged at the proteomic level, suggesting that post-transcriptional mechanisms may play a role in regulating the breast tumor microenvironment (46). Additionally, it has recently been reported that Siglec-7 may interact with PSGL-1 on chronic lymphocytic leukemia B cells, which is known to be one of VISTA interaction pathways, hinting at a potential negative feedback regulatory mechanism between Siglec-7 and VISTA in the mammary microenvironment (29, 67, 68). The correlation with A2AR was further corroborated in the bulk of TCGA samples. However, no significant association was observed in METABRIC patients. In contrast to our in-house cohort, Siglec-7 expression was positively correlated with VISTA in both METABRIC and TCGA datasets. Fascinatingly, while no associations were detected at the bulk expression level, intracellular analysis in METABRIC cohort identified an upregulation of A2AR in CD8+ and CD4+ cells from patients with elevated Siglec-7 expression, reinforcing the potential synergy effect between these two molecules. Furthermore, Siglec-7 expression was positively correlated with the upregulation of a distinct panel of inhibitory molecules known to contribute to immune evasion in breast cancer, including PD-1, PD-L1, CTLA-4, TIM-3, and LAG-3 (69). Siglec-7 was found to be significantly overexpressed on tumor-infiltrating CD8+ T cells within colon cancer tissues and ovarian cancer ascites, associated with the presence of exhaustion markers PD-1 and LAG-3. Antibodies targeting Siglec-7-ligand interactions resulted in a marked enhancement of T-cell cytotoxicity in both *in vivo* and *in vitro* models, thereby emphasizing Siglec-7 potential as a key immunotherapeutic target and its critical value in combinatory strategies (30). Additionally, CD4+ T cells displayed a distinct profile of inhibitory checkpoints in Siglec-7 high patients. The same previous study reported that CD4+ T cells, but not CD8+ T cells from peripheral blood, express inhibitory Siglecs, including Siglec-7, highlighting a differential expression pattern between these T cell subsets that may influence their molecular profiles (30).

To further comprehend the Siglec-7-mediated immunoregulatory network in breast cancer patients, we expanded our investigation to encompass the entire SIGLEC axis, seeking to elucidate potential synergistic or compensatory interactions among SIGLEC family members within the TME. Our analysis revealed a distinct SIGLEC expression signature in Siglec-7^HIGH^ breast tumors, marked by significant upregulation of various SIGLECs, including Siglec-5, -8, -9, and -14. Of particular interest was the cohort-specific regulation of Siglec-6, which was upregulated in TCGA patients with an elevated Siglec-7 expression profile, while downregulated in METABRIC. Conversely, Siglec-4 was consistently downregulated across both cohorts, while Siglec-15 showed a non-significant upregulation in Siglec-7^HIGH^ tumors. As we previously demonstrated, Siglec-7^HIGH^ patients exhibited significant enrichment in pathways related to sialic acid binding and metabolism, presumably associated with the upregulation of sialoglycan ligands on tumor cells, potentially driven by hypersialylation (21). This, in turn, may further strengthen the immunosuppressive SIGLEC-mediated network within the breast TME, potentially explaining the observed associations between Siglec-7 and other SIGLEC family members. Indeed, Siglec-5 was recently characterized as an inhibitor of T cell activation, and combining its targeting with Siglec-14 and Tamoxifen hormone therapy was suggested to enhance anti-tumor immunity in an *in vitro* breast cancer model (70, 71). Similarly, Siglec-15 was found to be upregulated in breast tumors and was reported to play a pivotal role in suppressing antigen-specific T cell responses, particularly in breast cancer bone metastasis (72). In contrast, Siglec-8 protein levels were enriched in higher-grade and ER-positive tumors, but significantly decreased in TNBC molecular subtype, suggesting a hormonally regulated, subtype-specific pattern in breast cancer patients (73). Furthermore, Siglec-7/Siglec-9 sialic acid-mediated interactions were associated with poor prognosis in pancreatic ductal adenocarcinoma, while elevated Siglec-6/Siglec-7 co-expression similarly correlated with reduced overall and disease-specific survival in bladder cancer (31, 57). SIGLECs are predominantly expressed on immune cells, with certain members restricted to specific cell types, and they mainly regulate immune responses through either inhibitory (ITIM) or activating (ITAM) signaling pathways (24). It is important to note that Siglec-6 is primarily expressed on B cells and placental trophoblasts, rather than on T cells, emphasizing its distinct cellular distribution (74). In our analysis, we demonstrated that elevated Siglec-7 expression in breast cancer patients was associated with a reduction in B cell infiltration. This finding may explain the observed negative correlation between Siglec-7 and Siglec-6 expression in METABRIC patients, suggesting possible cohort-specific regulatory mechanisms between these molecules within the breast TME. In contrast to ITIM/ITAM-mediated SIGLECs, Siglec-1 and Siglec-4 operate through non-tyrosine-based signaling, distinguishing their immune regulation within the SIGLEC family (75). It is worth highlighting that Siglec-4, also known as Myelin-Associated Glycoprotein (MAG), plays a pivotal role in neural myelin biology (76), underscoring its distinct functional profile compared to Siglec-7. Moreover, Siglec-4 downregulation in lung adenocarcinoma patients was associated with poor overall survival (77), suggesting a potential protective role against tumor progression and contrasting with the tumor-promoting role of Siglec-7 observed in our cohorts. Building on these observations, we hypothesize a negative regulatory feedback loop between Siglec-7 and Siglec-4 expression, potentially driving aggressive breast cancer phenotypes by sustaining an immunosuppressive TME. Taken together, these findings highlight the intricate nature of SIGLEC interactions in breast cancer, underscoring the need for further investigation into their role within the mammary tumor sialome. Moreover, our results showed that Siglec-7 expression is associated with the upregulation of signatures linked to compromised antitumor responses and the enrichment of immune escape pathways. These findings further underscore the immunosuppressive role of Siglec-7 in immune evasion, emphasizing its potential as a valuable biomarker for immune dysregulation in breast cancer. Indeed, it has been reported that Siglec-7-positive T cells show reduced proliferation in response to TCR stimulation and express elevated levels of inhibitory markers compared to Siglec-7-negative T cells, further highlighting the association between Siglec-7 and T cell exhaustion (30). Additionally, recent research has shown that the combination of Anti-Siglec-7 antibodies with anti-PD-1 further enhances NK cell-mediated cytotoxicity against ovarian cancer cell lines *in vitro* (33).

Our findings also underscored the significant link between high Siglec-7 expression and resistance to conventional breast cancer treatments, particularly chemotherapy and hormonotherapy. This highlights Siglec-7 as a promising target for precision medicine, enabling tailored treatment strategies to optimize response rates. Although the direct role of Siglec-7 in cancer therapy resistance has not been previously documented, studies have investigated the involvement of Siglec ligands, particularly hypersialylation, in mediating resistance mechanisms (20). In ovarian carcinoma, hypersialylation driven by overexpression of ST3GAL1 not only contributes to paclitaxel chemotherapy resistance but also enhances tumorigenicity (78). In addition, this mechanism upregulates epithelial–mesenchymal transition (EMT) in ovarian cancer cells, thereby promoting a more resistant phenotype (79). Furthermore, reactive oxygen species (ROS) pathways have been documented as key contributors to drug resistance in response to chemotherapeutic treatments (80). Notably, breast tumors with elevated levels of hypoxia-inducible factors are associated with resistance to various treatments, including conventional chemotherapy, targeted therapies, and endocrine therapy (81, 82). All the mechanisms outlined above were found to be significantly enriched in patients with high Siglec-7 expression, thus contributing to the understanding of their resistance profile. Of note, it was demonstrated that hypoxic stress induced an elevation in Siglec-7 sialoglycan ligands on a distinct group of NK cells isolated from PBMCs, while no alteration in the expression of Siglec-7/9 receptors was observed (83). Additionally, endocrine and hormonal imbalances are major contributors to breast cancer resistance, particularly in triple-negative and HER2-enriched subtypes (84–86), where elevated Siglec-7 expression has been observed. Following the same rationale, to assess the immunotherapy response and Siglec-7 expression, two parameters were analyzed, namely the Immunophenoscore (IPS) and EcoTyper. In fact, IPS represents a robust predictor of immunotherapy response, particularly to anti-CTLA-4 and anti-PD-1 immunotherapies. It is derived from algorithms that integrate key immunophenotypes and tumor escape mechanisms associated with immunotherapy resistance. Notably, a high IPS has been linked to a more favorable prognosis and improved outcomes with immunotherapy (34). Similarly, EcoTyper, a machine learning framework, offers promising potential in predicting immunotherapy response by analyzing the predominant ecotypes within the tumor microenvironment (44). Our analysis revealed that elevated Siglec-7 expression is significantly associated with a lower Immunophenoscore and a reduced abundance of CE9 and CE10 ecotypes, two critical factors showing a trend to favorable immunotherapy responses. These findings suggest that Siglec-7 may serve as a potential key target for immunotherapy, either as a monotherapy or in combinatory regimens, in patients exhibiting resistance to current therapeutic approaches. In the context of immunotherapy response, elevated Siglec-7 expression in intratumoral macrophages has been proposed as a novel predictive biomarker for the efficacy of immunotherapy in metastatic colorectal cancer patients (55). Furthermore, during Bacillus Calmette-Guerin (BCG) immunotherapy in bladder adenocarcinoma, Siglec-7 expression, along with Siglec-6, was associated with the suppression of antitumor immunity, thereby limiting the efficacy of this treatment and contributing to cancer recurrence (57).

Collectively, our study suggests that Siglec-7 could be a pivotal immune checkpoint in the pathophysiology of breast cancer, offering a potential target to address immune escape and therapy resistance in this disease.

## 5 Conclusion

In this study, we comprehensively explored the expression patterns and potential role of the promising glycoimmune checkpoint Siglec-7, elucidating its clinical, immunological, and therapeutic relevance in breast cancer patients. To the best of our knowledge, this is the first report offering novel insights into Siglec-7 transcripts in breast cancer, uncovering its potential synergistic action with inhibitory immune checkpoints, particularly A2AR. While this study provides valuable findings, we acknowledge its limitations, notably the small size of our in-house cohort. Nonetheless, our research lays the foundation for future proteomic and clinical investigations aimed at exploring the immune-related signatures of Siglec-7 across various molecular subtypes of breast cancer in patients, potentially paving the way to surmount immune evasion and improve treatment outcomes in this malignancy.

## Data Availability

The datasets presented in this study can be found in online repositories. The names of the repository/repositories and accession number(s) can be found in the article/**Supplementary Material**.

## Supplementary material

Supplementary Figure 1 Association between Siglec-7 gene expression, breast malignancy, and clinicopathological features in our in-house breast cancer cohort (n= 45). Siglec-7 transcript levels were measured using RT-PCR, with expression normalized to β-actin and analyzed relative to matched control tissues, presented as mRNA expression log^2^(^2^^(-ΔΔCt)). **(G, H, I, J, K)** No significant association was observed between Siglec-7 transcript levels and patient age, Ki-67 status, or TNM classification (Age: p = 0.2302, Ki-67: p = 0.1926, T: p = 0.4785, N: p = 0.2875, M: p = 0.9696). A p-value of less than 0.05 was considered statistically significant, with ‘ns’ indicating no statistical significance.

Supplementary Figure 2 Elevated Siglec-7 transcript levels correlated with unfavorable clinicopathological outcomes in patients with invasive breast carcinoma, based on RNA-seq and microarray data from the TCGA (n = 1070) and METABRIC (n = 1980) cohorts. Log2 normalization was applied to the raw data counts from TCGA for visualization. **(N, V)** Siglec-7 expression was not related to patients age in TCGA and METABRIC cohorts. **(O)** Siglec-7 expression was upregulated in both HER2+ and TNBC aggressive molecular subtypes compared to Luminal subtypes. **(P, Q, R, S, T, U)** No significant associations were found between Siglec-7 transcript levels and TNM classification, histological types, tumor stage, or survival in the TCGA dataset. *p < 0.01, **p < 0.001, ***p < 0.0001, and ns indicates no significance.

Supplementary Figure 3 Siglec-7 was linked to gene expression variations and was associated with signaling pathways and biological functions involved in breast cancer pathogenesis, as identified through differential gene and Gene Set Enrichment Analysis. **(D)** GSEA plots reveal significant differences in predefined gene sets, highlighting pathways essential for breast cancer development, progression, and aggressiveness, with both positive and negative enrichments observed in METABRIC Siglec-7HIGH cluster. **(E)** The bubble plot illustrates additional biological processes linked to cancer proliferation, invasion, angiogenesis, and metastasis in METABRIC Siglec-7HIGH cluster using molecular signatures from Hallmark, Ontology, and Curated databases. Terms with a nominal p-value < 0.05 and a false discovery rate (FDR) < 0.25 are considered statistically significant. NES stands for Normalized Enrichment Score, GO refers to Gene Ontology and 'ns' on GSEA plots indicates non-significance.

Supplementary Figure 4 Patients with elevated Siglec-7 expression were associated with a more immune-infiltrated tumor microenvironment and a positive enrichment of major immune function pathways. Estimate, TIDE, and GSEA analyses were used to assess the computed scores. **(E, F)** GSEA and bar plots consistently illustrate positive enrichment of key immune biological processes, as observed in the METABRIC cohort, in Siglec-7 high patients from TCGA dataset. Statistical significance was determined at p < 0.05 and FDR < 0.25.

Supplementary Figure 6 Siglec-7 overexpression correlated with the upregulation of a distinct inhibitory immune checkpoint profile across the tumor microenvironment and immune effector cells in breast cancer patients. **(E)** Siglec-7 expression shows a significant positive correlation with inhibitory immune checkpoints and pro-tumoral molecules in both TCGA and METABRIC cohorts. **(F)** Significant positive correlations were observed between Siglec-7 expression and various SIGLEC family members, alongside negative correlations with SIGLEC4 (r = –0.14; p < 0.0001, TCGA) and SIGLEC6 (r = –0.17; p < 0.0001, METABRIC). Spearman correlation coefficient was used to evaluate associations. **p < 0.01, ***p < 0.001, ****p < 0.0001, and 'ns' for no significant difference.

Supplementary Figure 7 High Siglec-7 expression in breast tumors was strongly linked to impaired antitumoral response signatures. TIDE, Z-score, and GSEA analyses were employed. **(D)** Siglec-7 mRNA expression is positively correlated with signatures associated with T-cell dysfunction and immune escape in both TCGA and METABRIC cohorts. Spearman correlation coefficient was used to evaluate associations. ****p < 0.0001, and 'ns' for no significant difference.

Supplementary Figure 8 Patients exhibiting high Siglec-7 expression demonstrated resistance to conventional breast cancer therapies and limited response to immunotherapy. **(E)** Immunophenoscore (IPS) analysis according to Siglec-7 expression profile in METABRIC patients. **(F)** High Siglec-7 expression is correlated with significant enrichment of pathways related to cancer therapy resistance in the METABRIC cohort. **(G)** Elevated Siglec-7 transcripts correlate with a decrease in the relative abundance of CE10 ecotype in METABRIC patients. The red color denotes Siglec-7HIGH group, and blue represents Siglec-7LOW group, and blue represents Siglec-7LOW group. Significance was established using p < 0.05, with *p < 0.05, **p < 0.01, and 'ns' for non-significant results.
