# Supplementary figures and images for: Elevated Siglec-7 expression correlates with adverse clinicopathological, immunological, and therapeutic response signatures in breast cancer patients
Source: Front Immunol. 2025 Jun 6;16:1573365. doi: 10.3389/fimmu.2025.1573365 (PMC12179189; doi:10.3389/fimmu.2025.1573365)

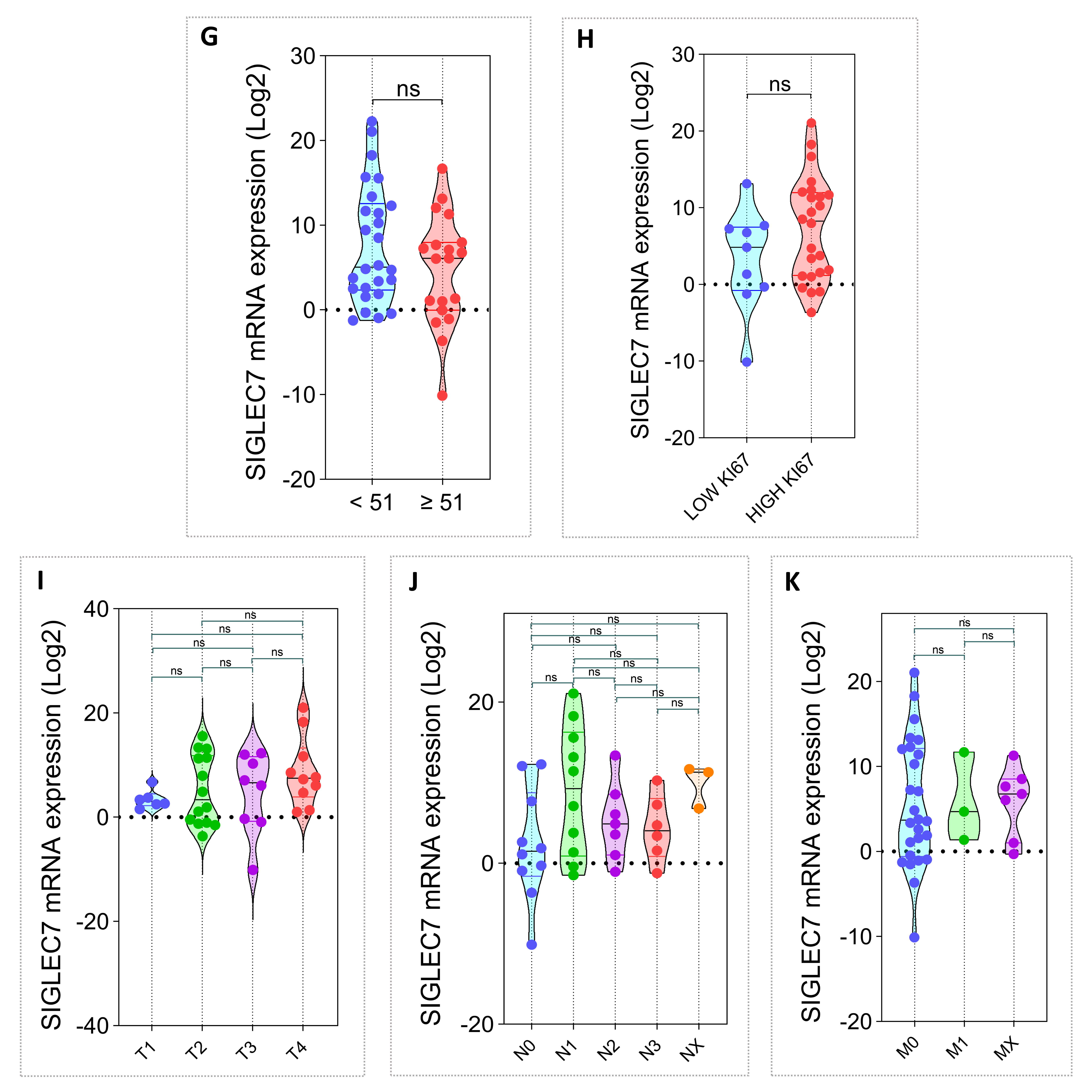

Supplement: Supplementary Figure 1 — Association between Siglec-7 gene expression, breast malignancy, and clinicopathological features in our in-house breast cancer cohort (n= 45). Siglec-7 transcript levels were measured using RT-PCR, with expression normalized to β-actin and analyzed relative to matched control tissues, presented as mRNA expression log2(2^(-ΔΔCt)). (G, H, I, J, K) No significant association was observed between Siglec-7 transcript levels and patient age, Ki-67 status, or TNM classification (Age: p = 0.2302, Ki-67: p = 0.1926, T: p = 0.4785, N: p = 0.2875, M: p = 0.9696). A p-value of less than 0.05 was considered statistically significant, with ‘ns’ indicating no statistical significance. [file Image1.tif]

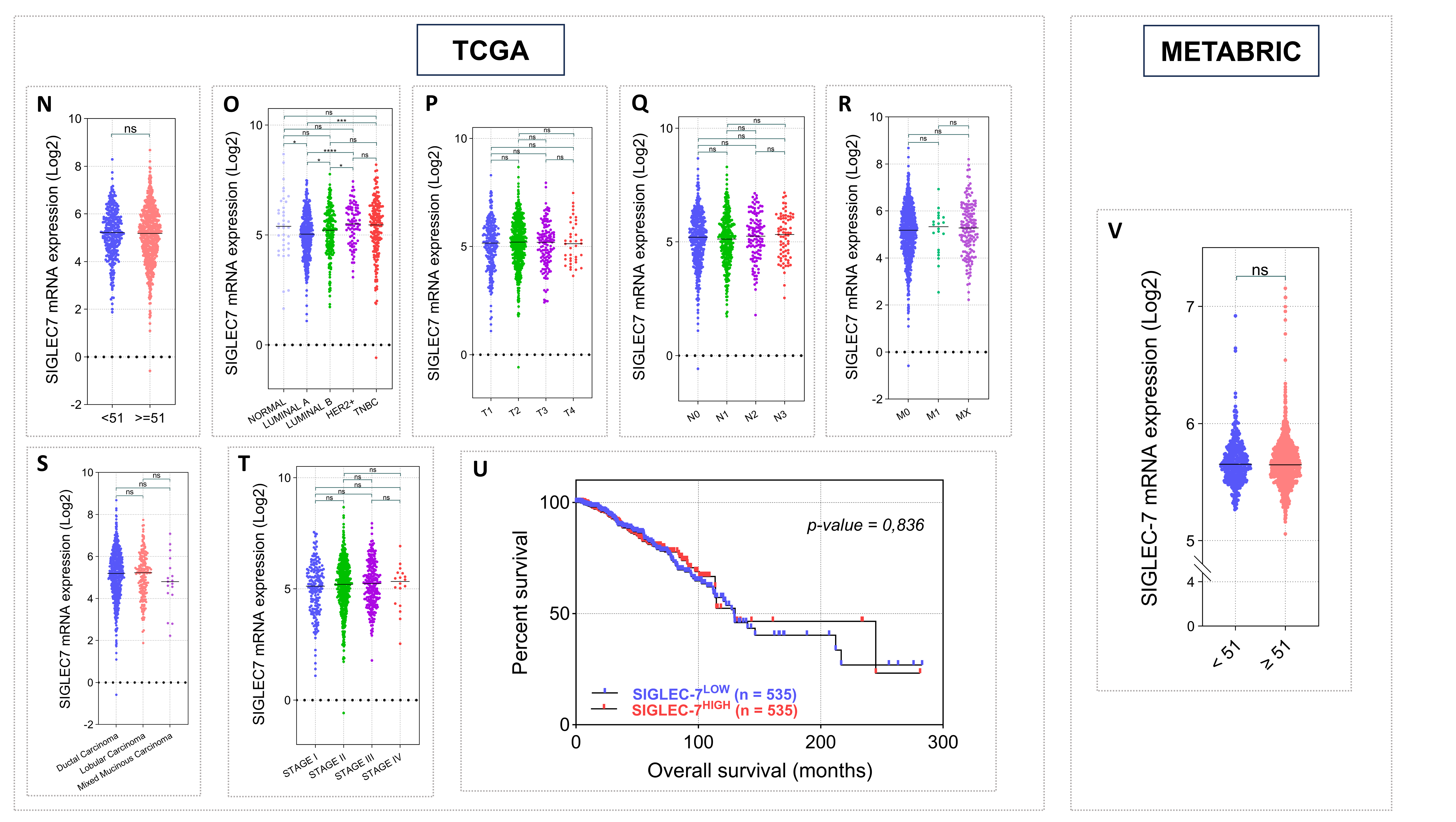

Supplement: Supplementary Figure 2 — Elevated Siglec-7 transcript levels correlated with unfavorable clinicopathological outcomes in patients with invasive breast carcinoma, based on RNA-seq and microarray data from the TCGA (n = 1070) and METABRIC (n = 1980) cohorts. Log2 normalization was applied to the raw data counts from TCGA for visualization. (N, V) Siglec-7 expression was not related to patients age in TCGA and METABRIC cohorts. (O) Siglec-7 expression was upregulated in both HER2+ and TNBC aggressive molecular subtypes compared to Luminal subtypes. (P, Q, R, S, T, U) No significant associations were found between Siglec-7 transcript levels and TNM classification, histological types, tumor stage, or survival in the TCGA dataset. *p < 0.01, **p < 0.001, ***p < 0.0001, and ns indicates no significance. [file Image2.tif]

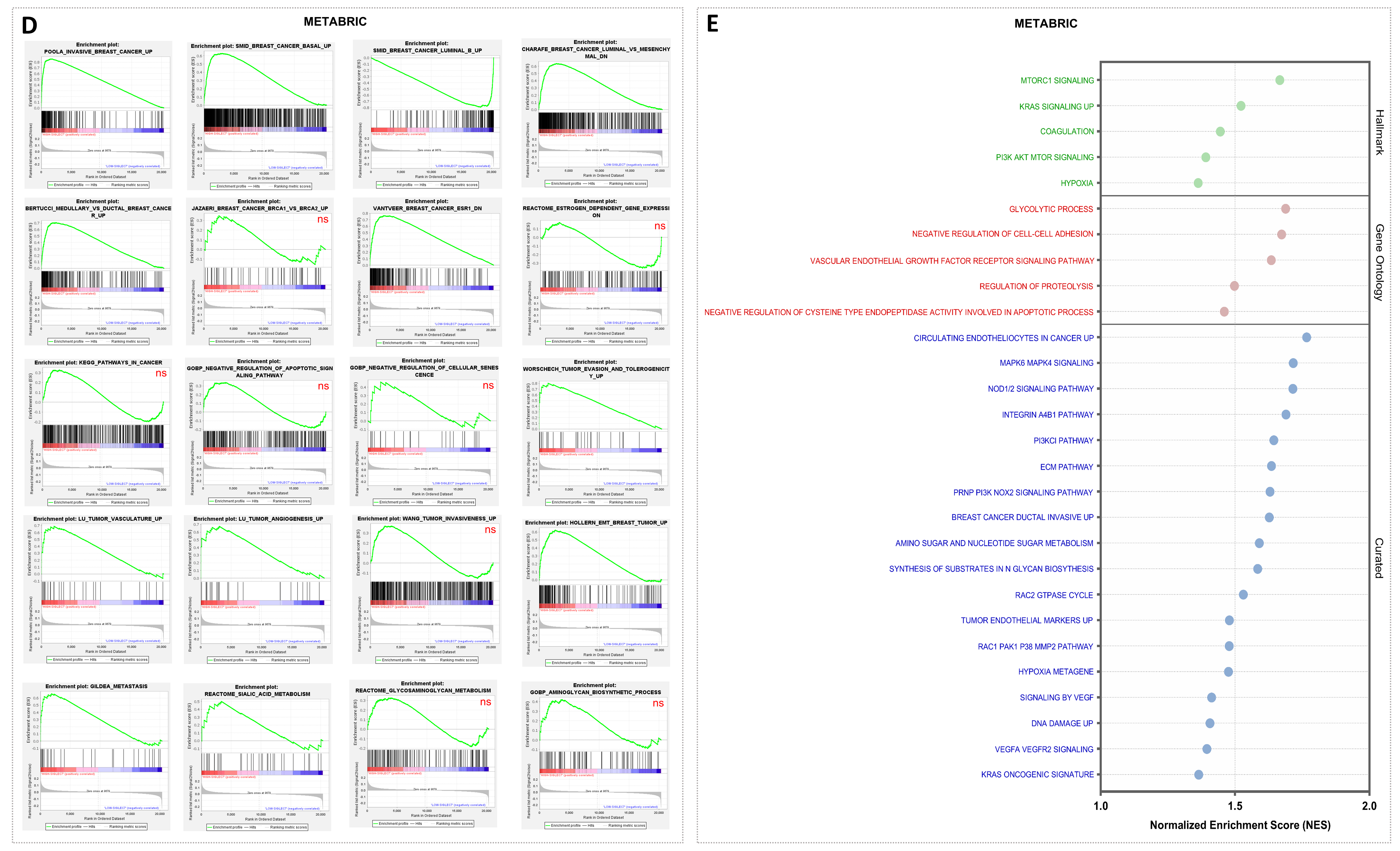

Supplement: Supplementary Figure 3 — Siglec-7 was linked to gene expression variations and was associated with signaling pathways and biological functions involved in breast cancer pathogenesis, as identified through differential gene and Gene Set Enrichment Analysis. (D) GSEA plots reveal significant differences in predefined gene sets, highlighting pathways essential for breast cancer development, progression, and aggressiveness, with both positive and negative enrichments observed in METABRIC Siglec-7HIGH cluster. (E) The bubble plot illustrates additional biological processes linked to cancer proliferation, invasion, angiogenesis, and metastasis in METABRIC Siglec-7HIGH cluster using molecular signatures from Hallmark, Ontology, and Curated databases. Terms with a nominal p-value < 0.05 and a false discovery rate (FDR) < 0.25 are considered statistically significant. NES stands for Normalized Enrichment Score, GO refers to Gene Ontology and 'ns' on GSEA plots indicates non-significance. [file Image3.tif]

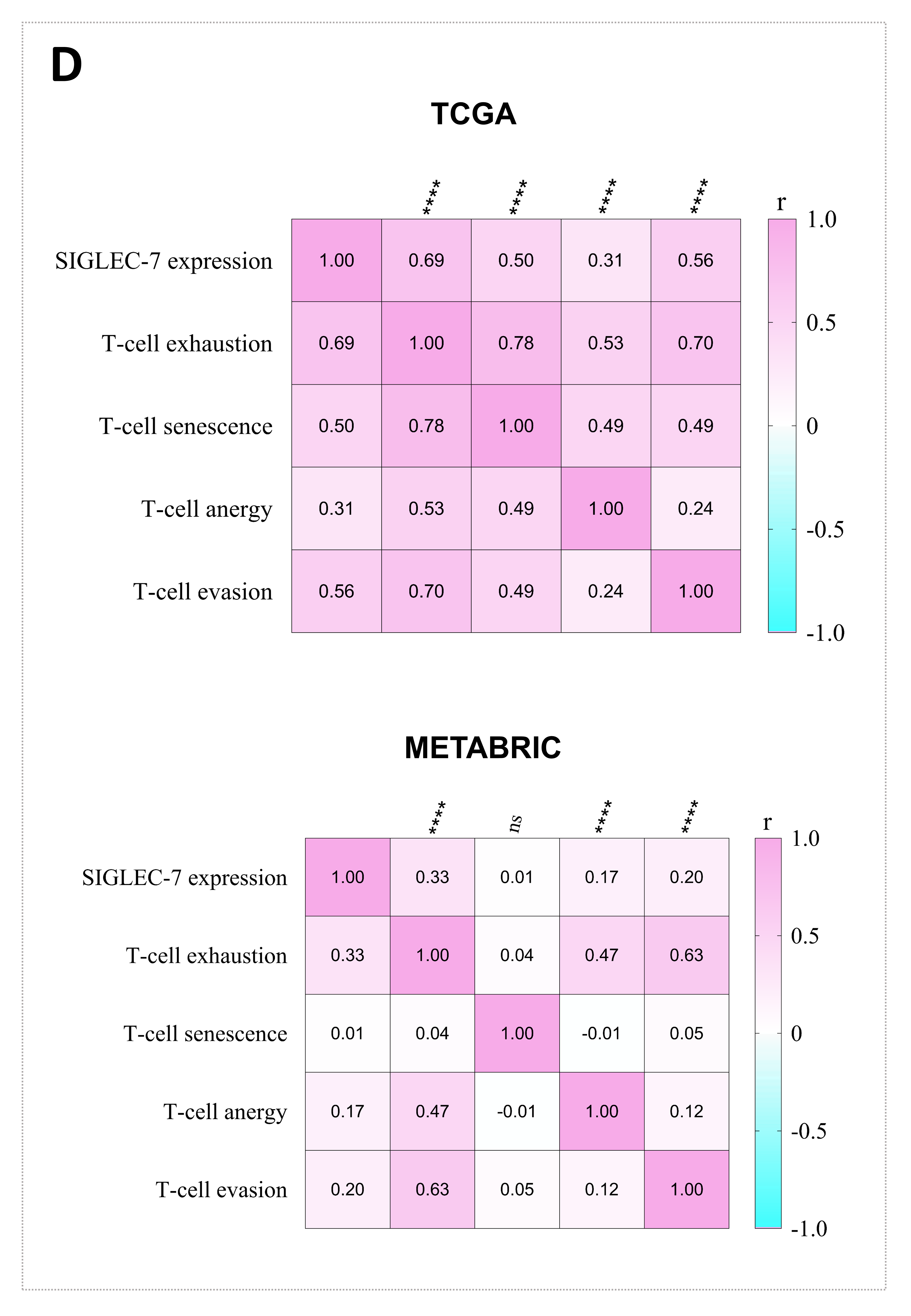

Supplement: Supplementary Figure 7 — High Siglec-7 expression in breast tumors was strongly linked to impaired antitumoral response signatures. TIDE, Z-score, and GSEA analyses were employed. (D) Siglec-7 mRNA expression is positively correlated with signatures associated with T-cell dysfunction and immune escape in both TCGA and METABRIC cohorts. Spearman correlation coefficient was used to evaluate associations. ****p < 0.0001, and 'ns' for no significant difference. [file Image7.tif]

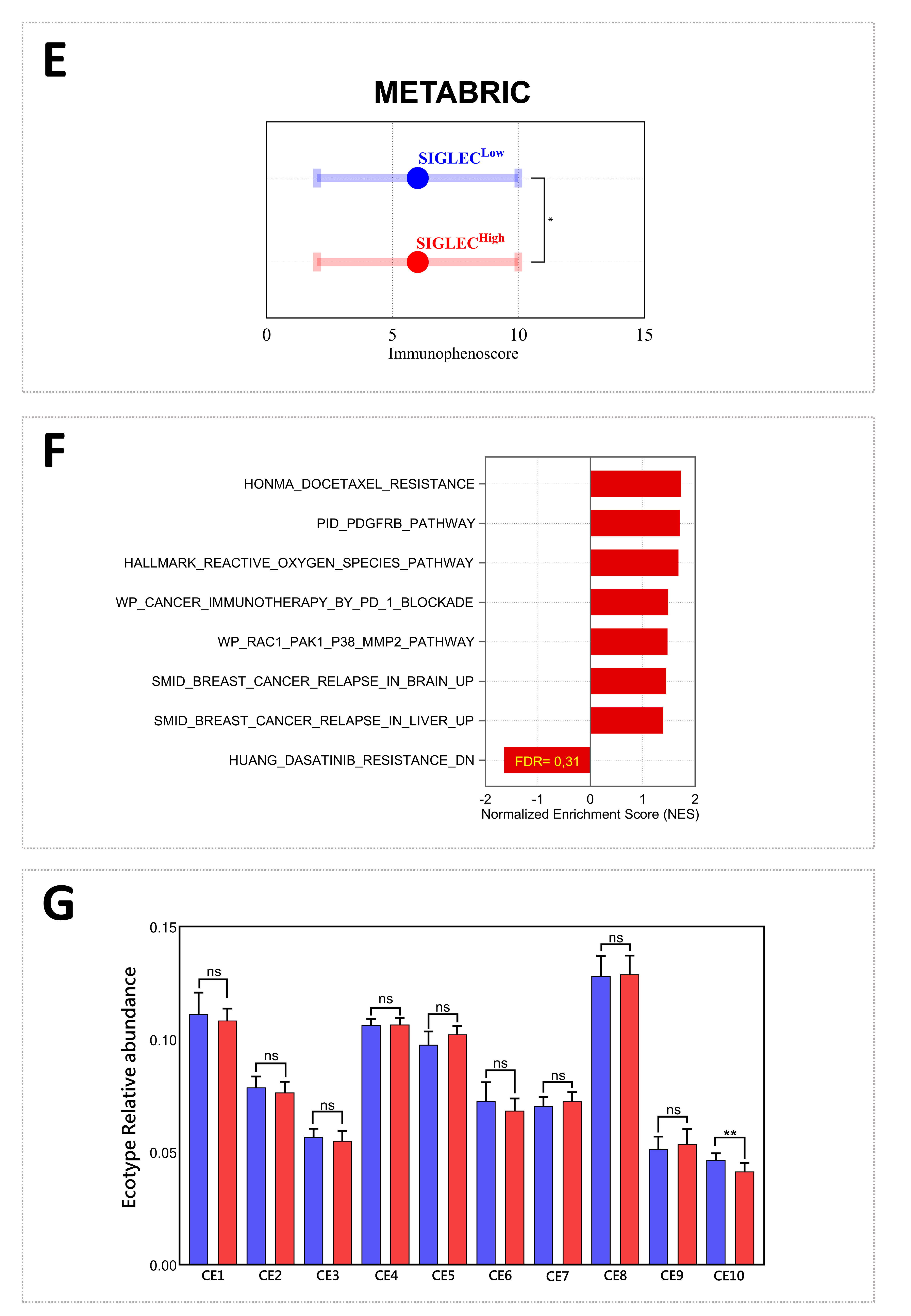

Supplement: Supplementary Figure 8 — Patients exhibiting high Siglec-7 expression demonstrated resistance to conventional breast cancer therapies and limited response to immunotherapy. (E) Immunophenoscore (IPS) analysis according to Siglec-7 expression profile in METABRIC patients. (F) High Siglec-7 expression is correlated with significant enrichment of pathways related to cancer therapy resistance in the METABRIC cohort. (G) Elevated Siglec-7 transcripts correlate with a decrease in the relative abundance of CE10 ecotype in METABRIC patients. The red color denotes Siglec-7HIGH group, and blue represents Siglec-7LOW group, and blue represents Siglec-7LOW group. Significance was established using p < 0.05, with *p < 0.05, **p < 0.01, and 'ns' for non-significant results. [file Image8.tif]
