# Supplementary material for: Elevated Siglec-7 expression correlates with adverse clinicopathological, immunological, and therapeutic response signatures in breast cancer patients
Source: Front Immunol. 2025 Jun 6;16:1573365. doi: 10.3389/fimmu.2025.1573365 (PMC12179189; doi:10.3389/fimmu.2025.1573365)
Supplement: Supplementary file 8 [file Table1.docx]

**Supplementary Table 1.** Siglec-7 gene expression across different clinicopathological features in our in-house cohort.

| Clinicopathological parameters | Cases (%) (n=45) | *p-*value |
| --- | --- | --- |
| Age | | |
| - < 51 - >= 51 | 26 (57.78%)  19 (42.22%) | 0.2302 |
| Histological grade | | |
| - I - II - III | 3 (6.66%)  21 (46.66%)  21 (46.66%) | 0.0603 |
| Molecular subtype | | |
| - Luminal A - Luminal B - HER2 - TNBC | 13 (28.88%)  12 (26.67%)  8 (17.78%)  12 (26.67%) | 0.0002 |
| ER status | | |
| - ER+ - ER- | 25 (55.56%)  20 (44.44%) | <0.0001 |
| PR status | | |
| - PR+ - PR- | 25 (55.56%)  20 (44.44%) | 0.0002 |
| HER2 status | | |
| - HER2+ - HER2- | 14 (31.11%)  31 (68.89%) | 0.2375 |
| T classification | | |
| - T1 - T2 - T3 - T4 - Missing data | 6 (13.33%)  14 (31.11%)  8 (17.78%)  10 (22.22%)  7 (15.56) | 0.4785 |
| N classification | | |
| - N0 - N1 - N2 - N3 - NX - Missing data | 10 (22.22%)  10 (22.22%)  7 (15.56%)  6 (13.33%)  3 (6.67%)  9 (20%) | 0.2875 |
| M classification | | |
| - M0 - M1 - MX - Missing data | 26 (57.78%)  3 (6.67%)  7 (15.55%)  9 (20%) | 0.9696 |
| Ki-67 proliferation index | | |
| - Low-Ki-67 - High-Ki-67 - Missing data | 9 (20%)  24 (53.33%)  12 (26.67) | 0.1926 |

*HER-2: human epidermal growth factor receptor-2, TNBC: triple negative breast cancer, ER: estrogen receptor and PR: progesterone receptor. *Statistical analyses were conducted on the parameters highlighted in bold.*
