# Supplementary material for: Elevated Siglec-7 expression correlates with adverse clinicopathological, immunological, and therapeutic response signatures in breast cancer patients
Source: Front Immunol. 2025 Jun 6;16:1573365. doi: 10.3389/fimmu.2025.1573365 (PMC12179189; doi:10.3389/fimmu.2025.1573365)
Supplement: Supplementary file 9 [file Table2.docx]

**Supplementary Table 2.** Association of Siglec-7 gene expression with clinicopathological parameters in TCGA cohort.

| Clinicopathological parameters | Cases (%) (n=1070) | *p-*value |
| --- | --- | --- |
| Age | | |
| - < 51 - >= 51 | 325 (30.37%)  745 (69.62%) | 0.2912 |
| PAM50 Molecular Subtype | | |
| - Luminal A - Luminal B - HER2 - TNBC - Normal-like - Missing data | 310 (28.97%)  242 (22.62%)  146 (13.65%)  229 (21.40%)  136 (12.71%)  7 (0.65%) | <0.0001 |
| PR status | | |
| - PR+ - PR- - Missing data | 664 (62.06%)  329 (30.75%)  77 (7.19%) | 0.0397 |
| ER status | | |
| - ER+ - ER- - Missing data | 766 (71.59%)  230 (21.50%)  74 (6.91%) | <0.0001 |
| HER2 status | | |
| - HER2+ - HER2- - Missing data | 152 (14.20%)  852 (79.63%)  66 (6.17%) | 0.0119 |
| Molecular Subtype | | |
| - Luminal A - Luminal B - HER2 - TNBC - Normal-like - Missing data | 530 (49.54%)  206 (19.25%)  85 (7.94%)  189 (17.66%)  36 (3.36%)  24 (2.25%) | <0.0001 |
| Histological type | | |
| - Ductal carcinoma - Lobular carcinoma - Mixed Mucinous carcinoma - NOS, other and missing data | 769 (71.87%)  201 (18.78%)  17 (1.59%)  83 (7.76%) | 0.4615 |
| Tumor stage | | |
| - Stage I - Stage II - Stage III - Stage IV - Stage X and missing data | 180 (16.82%)  607 (56.73%)  246 (22.99%)  18 (1.68%)  19 (1.77%) | 0.6829 |
| T classification | | |
| - T1 - T2 - T3 - T4 - TX | 275 (25.71%)  617 (57.66%)  137 (12.80%)  38 (3.55%)  3 (0.28) | 0.7488 |
| N classification | | |
| - N0 - N1 - N2 - N3 - NX | 508 (22.22%)  349 (22.22%)  118 (15.56%)  75 (13.33%)  20 (6.67%) | 0.5037 |
| M classification | | |
| - M0 - M1 - MX | 890 (83.18%)  20 (1.87%)  160 (14.95%) | 0.4786 |

*HER-2: human epidermal growth factor receptor-2, TNBC: triple negative breast cancer, ER: estrogen receptor and PR: progesterone receptor. *Statistical analyses were conducted on the parameters highlighted in bold.*
