# Supplementary material for: Elevated Siglec-7 expression correlates with adverse clinicopathological, immunological, and therapeutic response signatures in breast cancer patients
Source: Front Immunol. 2025 Jun 6;16:1573365. doi: 10.3389/fimmu.2025.1573365 (PMC12179189; doi:10.3389/fimmu.2025.1573365)
Supplement: Supplementary file 10 [file Table3.docx]

**Supplementary Table 3.** Clinicopathological associations of Siglec-7 gene expression in the METABRIC cohort.

| Clinicopathological parameters | Cases (%) (n=1980) | *p-*value |
| --- | --- | --- |
| Age | | |
| - < 51 - >= 51 | 470 (23.74%)  1510 (76.26%) | 0.3564 |
| Histological grade | | |
| - G1 - G2 - G3 - Missing data | 169 (8.53%)  771 (38.94%)  952 (48.09%)  88 (4.44%) | <0.0001 |
| Tumor stage | | |
| - 0 - 1 - 2 - 3 - 4 - Missing data | 12 (0.61%)  501 (25.30%)  825 (41.67%)  118 (5.96%)  10 (0.50%)  514 (25.96%) | 0.0088 |
| Histological type | | |
| - Tubular - Mucinous - Lobular - Ductal - Medullary - Mixed - Other and missing data | 21 (1.06%)  23 (1.16%)  146 (7.37%)  1491 (75.30%)  25 (1.26%)  211 (10.66%)  63 (3.19%) | <0.0001 |
| PAM50 Molecular Subtype | | |
| - Luminal A - Luminal B - HER2 - TNBC - Claudine-low - Normal-like - Missing data | 700 (35.35%)  475 (23.99%)  224 (11.31%)  209 (10.56%)  218 (11.01%)  148 (7.48%)  6 (0.30%) | <0.0001 |
| PR status | | |
| - PR+ - PR- | 1040 (52.52%)  940 (47.48%) | <0.0001 |
| ER status | | |
| - ER+ - ER- | 1506 (76.06%)  474 (23.94%) | <0.0001 |
| HER2 status | | |
| - HER2+ - HER2- | 247 (12.47%)  1733 (87.53%) | 0.5573 |
| NPI score category | | |
| - Excellent (≤ 2.4) - Good (2.4 - 3.4) - Moderate (3.4 - 5.4) - Poor (> 5.4) | 182 (9.19%)  498 (25.15%)  1101 (55.61%)  199 (10.05%) | <0.0001 |

*HER-2: human epidermal growth factor receptor-2, TNBC: triple negative breast cancer, ER: estrogen receptor and PR: progesterone receptor. *Statistical analyses were conducted on the parameters highlighted in bold.*
